# miR164g-*MsNAC022* acts as a novel module mediating drought response by transcriptional regulation of reactive oxygen species scavenging systems in apple

**DOI:** 10.1093/hr/uhac192

**Published:** 2022-08-30

**Authors:** Xiang Peng, Chen Feng, Yan-Tao Wang, Xiang Zhang, Yan-Yan Wang, Yue-Ting Sun, Yu-Qin Xiao, Ze-Feng Zhai, Xin Zhou, Bing-Yang Du, Chao Wang, Yang Liu, Tian-Hong Li

**Affiliations:** State Key Laboratories of Agrobiotechnology, Department of Pomology, College of Horticulture, China Agricultural University, Beijing 100193, China; State Key Laboratories of Agrobiotechnology, Department of Pomology, College of Horticulture, China Agricultural University, Beijing 100193, China; Laboratory of the Ministry of Agriculture, Agricultural Genomics Institute, Chinese Academy of Agricultural Sciences, Shenzhen 518120, China; State Key Laboratories of Agrobiotechnology, Department of Pomology, College of Horticulture, China Agricultural University, Beijing 100193, China; State Key Laboratories of Agrobiotechnology, Department of Pomology, College of Horticulture, China Agricultural University, Beijing 100193, China; State Key Laboratories of Agrobiotechnology, Department of Pomology, College of Horticulture, China Agricultural University, Beijing 100193, China; State Key Laboratories of Agrobiotechnology, Department of Pomology, College of Horticulture, China Agricultural University, Beijing 100193, China; State Key Laboratories of Agrobiotechnology, Department of Pomology, College of Horticulture, China Agricultural University, Beijing 100193, China; State Key Laboratories of Agrobiotechnology, Department of Pomology, College of Horticulture, China Agricultural University, Beijing 100193, China; State Key Laboratories of Agrobiotechnology, Department of Pomology, College of Horticulture, China Agricultural University, Beijing 100193, China; State Key Laboratories of Agrobiotechnology, Department of Pomology, College of Horticulture, China Agricultural University, Beijing 100193, China; State Key Laboratories of Agrobiotechnology, Department of Pomology, College of Horticulture, China Agricultural University, Beijing 100193, China; State Key Laboratories of Agrobiotechnology, Department of Pomology, College of Horticulture, China Agricultural University, Beijing 100193, China

## Abstract

Under drought stress, reactive oxygen species (ROS) overaccumulate as a secondary stress that impairs plant performance and thus severely reduces crop yields. The mitigation of ROS levels under drought stress is therefore crucial for drought tolerance. MicroRNAs (miRNAs) are critical regulators of plant development and stress responses. However, the complex molecular regulatory mechanism by which they function during drought stress, especially in drought-triggered ROS scavenging, is not fully understood. Here, we report a newly identified drought-responsive miRNA, miR164g, in the wild apple species *Malus sieversii* and elucidate its role in apple drought tolerance. Our results showed that expression of miR164g is significantly inhibited under drought stress and it can specifically cleave transcripts of the transcription factor *MsNAC022 in M. sieversii*. The heterologous accumulation of miR164g in *Arabidopsis thaliana* results in enhanced sensitivity to drought stress, while overexpression of *MsNAC022* in Arabidopsis and the cultivated apple line ‘GL-3’ (*Malus domestica* Borkh*.*) lead to enhanced tolerance to drought stress by raising the ROS scavenging enzymes activity and related genes expression levels, particularly *PEROXIDASE (MsPOD)*. Furthermore, we showed that expression of *MsPOD* is activated by MsNAC022 in transient assays. Interestingly, *Part1* (*P1*) region is the key region for the positive regulation of *MsPOD* promoter by MsNAC022, and the different *POD* expression patterns in *M. sieversii* and *M. domestica* is attributed to the specific fragments inserted in *P1* region of *M. sieversii*. Our findings reveal the function of the miR164g*-MsNAC022* module in mediating the drought response of *M. sieversii* and lay a foundation for breeding drought-tolerant apple cultivars.

## Introduction

Various abiotic stresses such as drought and high soil salinity inevitably accompany plant growth and development [1–4]. Among them, drought stress severely reduces crop yields by at least 40% worldwide, a proportion that continues to rise due to climate change [5, 6]. Apple (*Malus* sp.) is one of the most economically important fruit trees and is widely cultivated globally, but apple fruit quality and yield have dramatically declined with the more common occurrence of drought stress [3, 4]. Characterization of the molecular components and signaling pathways related to drought stress represents the first step toward improving drought resistance and developing efficient strategies for breeding drought-tolerant apple cultivars. *Malus sieversii* Roem*.,* an ancestral species of modern cultivated apples, has become a valuable resource for exploring drought response mechanisms and is the commonly used apple rootstock in some arid and semi-arid regions with exceptional drought stress tolerance [7–9]. However, little is currently known regarding how *M. sieversii* exhibits such prominent drought tolerance, which severely hampers our ability to breed drought-tolerant apple cultivars.

Drought responses in plants are characterized by reduced leaf water potential and lower turgor pressure, which result in stomatal closure and eventually influence cell growth and elongation [2, 10, 11]. When drought extends beyond a given duration, plant cells start to excessively accumulate reactive oxygen species (ROS), which leads to oxidative damage, including membrane peroxidation, protein denaturation, nucleic acid damage, and ultimately cell death [12, 13]. To counteract the oxidative burst caused by ROS accumulation, plants employ tightly controlled ROS scavenging systems composed of enzymatic and non-enzymatic antioxidants. The enzymatic pathways include superoxide dismutase (SOD), peroxidase (POD), catalase (CAT), ascorbate peroxidase (APX) and glutathione peroxidase (GPX), while the non-enzymatic system mainly comprises antioxidants, such as ascorbic acid (AsA), carotenoids, α-tocopherol, and glutathione. Enzyme activity and the contents of these reducing substances are positively correlated with plant resistance to abiotic stress such as drought and salt stress [14, 15]. Therefore, plants with higher drought tolerance are thought to be better equipped at engaging their ROS scavenging systems. For instance, *M. sieversii* exhibits higher POD and SOD activities under drought stress, suggesting that these two enzymes and the transcript levels of their encoding genes are differentially regulated in *M. sieversii* compared to other apple cultivars [14]. In model plants, *NAM, ATAF1/2,* and *CUC2* (NAC) transcription factors confer resistance to abiotic stress by regulating ROS scavenging systems [16–18]. For example, the *Arabidopsis thaliana* (Arabidopsis) NAC transcription factor JUNGBRUNNEN1 (JUB1), the expression of whose encoding gene is induced by hydrogen peroxide (H_2_O_2_), dampens intracellular H_2_O_2_ levels and enhances tolerance to various abiotic stresses by inducing its direct target *DEHYDRATION-RESPONSIVE ELEMENT BINDING PROTEIN 2A* (*DREB2A*) [17]. In rice (*Oryza sativa*), the stress-responsive NAC transcription factor SNAC3 confers drought tolerance through modulation of ROS [18]. Nevertheless, the role of NAC transcription factors in regulating ROS scavenging systems under drought stress remains to be explored in apple.

MicroRNAs (miRNAs) are endogenous small single-stranded non-coding RNAs that play vital regulatory roles in plant drought tolerance. Multiple miRNAs have been reported to cleave transcripts of several drought-responsive genes and hence prevent the accumulation of their encoded proteins [19–23]. For instance, drought stress substantially downregulates the expression of miR169a and miR169c, while the abundance of their target transcript from *NUCLEAR FACTOR Y A5* (*NF-YA5*) accumulates upon drought stress. Transgenic Arabidopsis plants overexpressing *NF-YA5* display enhanced drought resistance [24]. The abundance of miR165/166 and their target transcript *β-1,3-GLUCANASE 1* (*BG1*) also respond to drought stress. Lower *miR165/166* expression leads to a greater accumulation of *BG1* transcripts and thus abscisic acid (ABA) contents, ultimately enhancing drought tolerance in Arabidopsis [25].

The miR164 family was first identified as a class of miRNAs that function in plant growth and development in Arabidopsis, various *miR164* loci participate in the development of lateral root and shoot apical meristems, the establishment of the cotyledon boundary and floral organs, fruit ripening, and pathogen-induced and age-dependent cell death [26–34]. Roles for miR164 in abiotic stress, especially drought stress, have emerged in recent years. miRNA transcriptome analysis in maize (*Zea mays*) identified multiple drought-responsive miR164 isoforms [35]. In other plant species such as wheat (*Triticum aestivum*), populus (*Populus trichocarpa*) and Medicago (*Medicago truncatula*), drought stress downregulates the transcription of *miR164* genes [36–39]. Such regulation in different species for miR164 family members suggested that they might play crucial roles in drought responses. However, the functional role of miR164 in drought stress is poorly studied.

In the present study, we identified and characterized miR164g and its target transcript *MsNAC022* in *M. sieversii* from our previous high-throughput small-RNA and degradome sequencing [40]*.* A new member of the apple miR164 family, miR164g plays a crucial role in the drought response of *M. sieversii* miR164s (msi-miR164s). We show here that overexpression of *msi-miR164g* in Arabidopsis produces drought-sensitive seedlings, while overexpression of its target *MsNAC022* in Arabidopsis and the cultivated apple line ‘GL-3’ enhances drought resistance. Phenotypic analysis revealed that the activity of ROS scavenging enzymes and the expression of their encoding genes are prominently higher in *MsNAC022* overexpression plants. Furthermore, two insertions in the *MsPOD* promoter resulted in its greater transcriptional activation by MsNAC022, suggesting that improved ROS scavenging activity may contribute to the differences of drought tolerance between *M. sieversii* and *Malus domestica*.

## Results

### Identification of msi-miR164g and different expression patterns for *miR164* family members in *M. sieversii* under drought stress

**Figure 1:**
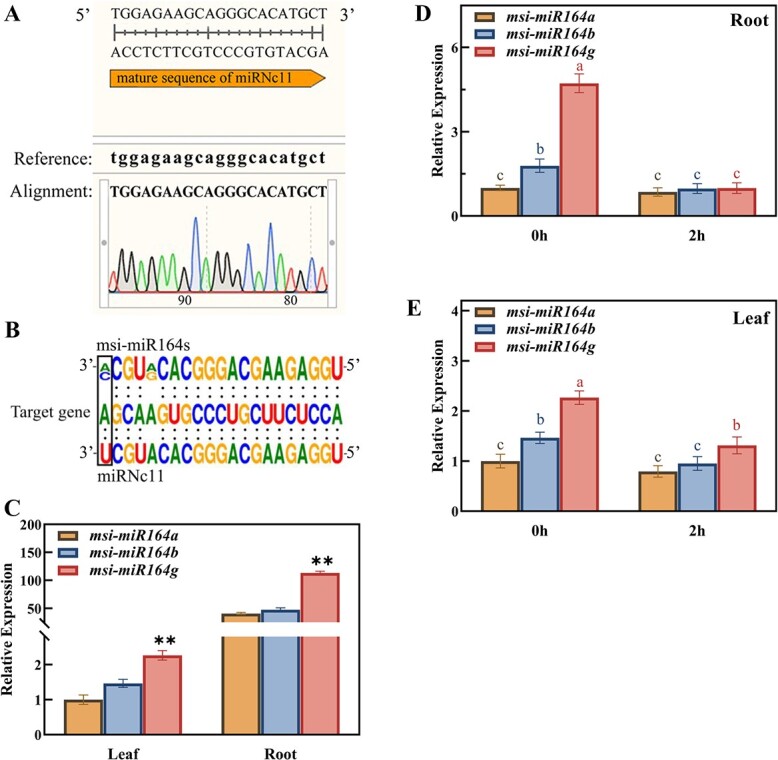
Identification of msi-miR164g and expression patterns of *miR164*s in *Malus sieversii* under drought stress. **A** Validation of the mature free miRNA for the newly predicted miRNc11. **B** Comparative analysis of miRNc11 and msi-miR164 family members. The mature sequences of miRNc11 and msi-miR164s were aligned to their target gene. **C** Tissue accumulation patterns of *miR164a/b/g* under normal growth conditions in *M. sieversii.* Data are shown as means ± standard deviation (SD) from three biological replicates. Asterisks indicate significant differences among members (^**^P < 0.01, based on Duncan’s multiple range test). **D**, **E** Expression levels of *msi-miR164a/b/g* in *M. sieversii* treated with 20% (w/v) PEG 6000. Data are shown as means ± SD from three biological replicates. Different letters indicate significant differences (*P* < 0.05, based on Duncan’s multiple range test).

*M. sieversii* is an ancestral species of modern apple cultivars that shows exceptional drought tolerance. To determine the candidate genes responsible for the drought resistance, we previously performed high-throughput small RNA sequencing and identified various small non-coding RNAs (snRNAs) and their targets in drought-exposed *M. sieversii* [40]. Here, we selected the novel miRNA namely miRNc11, which was differentially expressed under short-term drought stress treatments, for investigation of its regulatory role in the drought response. We conducted specific stem–loop reverse transcription PCR to validate the new predicted miRNc11 in *M. sieversii* and *M. domestica*. The identified free mature miRNA only exists in *M. sieversii* and aligned with miRNc11 exactly (Fig. 1A; Fig. S1, see online supplementary material). Sequence analysis also revealed that the miRNc11 mature sequence displays high sequence similarity to the msi-miR164 family, with only one nucleotide difference from msi-miR164a at nucleotide 21 in the 5′ end and two nucleotide differences from msi-miR164b/c/d/e/f at positions 17 and 21 in the 5′ end (Fig. S2, see online supplementary material). The difference between miRNc11 and msi-miR164a at position 21 in the 5′ end resulted in better alignment with the recognition sites in the target gene (Fig. 1B). We thus named this novel miRNc11 msi-miR164g, as the third mature sequence of msi-miR164 family. We also constructed a phylogenetic tree using the sequences of 126 *miR164* precursors from 36 plant species (Fig. S3, see online supplementary material). We determined that miR164s are conserved throughout the plant kingdom and that each plant species has 1–11 miR164 family members (Fig. S3B, see online supplementary material).

Quantitative real-time PCR of *msi-miR164a/b/g* revealed their high expression levels in roots relative to leaves under normal growth conditions. In addition, *msi-miR164g* expression was two to three times higher than that of *msi-miR164a* and *msi-miR164b*, indicating that *msi-miR164g* largely contributes to the function of the miR164 family in *M. sieversii* (Fig. 1C)*.* Promoter analysis of the *msi-miR164g* locus identified multiple *cis*-acting elements related to drought, salinity, ABA, and low temperature signaling, indicating that msi-miR164g may accumulate in response to multiple environmental stimuli (Table S1, see online supplementary material). To test whether *msi-miR164* responded to drought stress, we mimicked drought conditions using 20% (w/v) PEG 6000 treatment and examined the expression patterns of *msi-miR164a/b/g* in *M. sieversii*. The results showed that *msi-miR164g* expression is much more induced over the course of the treatment than that of *msi-miR164a/b* expression. In particular, *msi-miR164g* expression in roots and leaves were rapidly down-regulated at 2 h of drought treatment, indicating that *msi-miR164g* respond to drought stress (Fig. 1D, E).

### Msi-miR164g cleaves the transcript of target gene *MsNAC022*

The miR164 targets identified in other plant species belong to the *NAC* gene family [27, 30, 32, 39, 41]. To determine which candidate *NAC* gene msi-miR164g might target in apple, we performed an alignment search using the mature msi-miR164g sequence and the apple transcript database. The transcript for gene MD10G1198400 showed the best alignment with msi-miR164g, making it an excellent candidate target for msi-miR164g; we named this gene *MsNAC022* based on the amino acid sequence similarity (Fig. S4, see online supplementary material). Using the msi-miR164g sequence as a query against the Arabidopsis transcript database retrieved the same candidate *NAC* genes as ath-miR164 (Fig. S5, see online supplementary material), indicating that the miR164g*-NAC* module is conserved among plant species.

**Figure 2:**
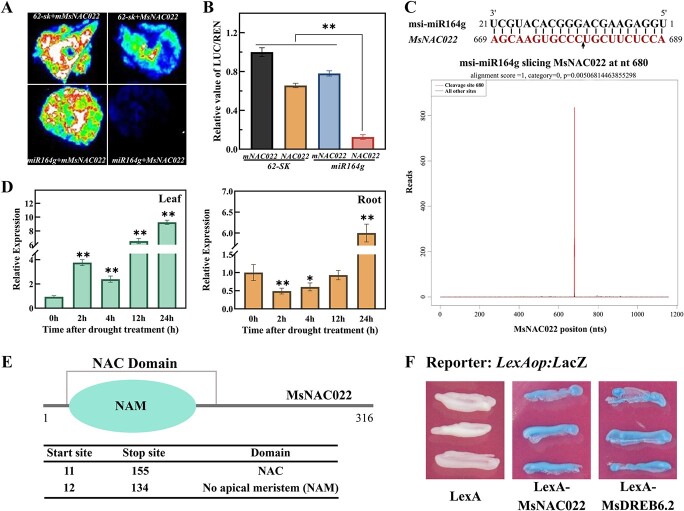
msi-miR164g targets transcripts encoding the NAC transcription factor MsNAC022. **A**, **B** Dual luciferase reporter assay to evaluate msi-miR164g effects on its target transcript *MsNAC022*. *mMsNAC022*, synonymous mutation of *MsNAC022* in the msi-miR164g cleavage sites. Renilla luciferase (*REN*) was used as a positive control. Data are shown as means ± SD from three biological replicates. Asterisks indicate significant differences (^**^P < 0.01, based on Duncan’s multiple range test). **C** msi-miR164g-directed cleavage sites of *MsNAC022* transcripts, as identified by degradome analysis with high-throughput 5’ RACE sequencing. The arrow indicates the msi-miR164g cleavage sites in the *MsNAC022* transcript. **D** Expression patterns of *MsNAC022* in *Malus sieversii* treated with 20% PEG 6000. Data are shown as means ± SD from three biological replicates. Asterisks indicate significant differences (^*^P < 0.05, ^**^P < 0.01, based on Duncan’s multiple range test). **E** Functional domains in MsNAC022. **F** Transcriptional activation activity assay of MsNAC022 in yeast strain EGY48. MsDREB6.2 was used as a positive control.

To ascertain the effect of msi-miR164g on the abundance of *MsNAC022* transcripts, we performed a dual luciferase-based miRNA sensor assay to qualitatively and quantitatively evaluate the cleavage of *MsNAC022* transcripts by msi-miR164g. The putative cleavage sites of msi-miR164g were located in the open reading frame of *MsNAC022*; we thus constructed a sensor vector *LUC*-*MsNAC022* that expresses the firefly luciferase (*LUC*) reporter gene cloned in-frame with the *MsNAC022* cleavage sites under the control of the cauliflower mosaic virus 35S promoter. We also generated a second sensor, *LUC*-*mMsNAC022*, with synonymous mutations in the cleavage sites. The transient infiltration of either sensor construct alone in *Nicotiana benthamiana* leaves resulted in strong relative LUC activity; by contrast, co-infiltration of the *LUC* sensors with a construct overexpressing *pre-msi-miR164g* dramatically reduced LUC activity from *LUC*-*MsNAC022*, but not from *LUC*-*mMsNAC022* harboring a mutation in the presumed *msi*-miR164g target site (Fig. 2A, B). These results were consistent with the notion that msi-miR164g targets the *MsNAC022* transcript.

To confirm the cleavage sites, we conducted sequencing of a degradome library with high-throughput 5′ rapid amplification of cDNA ends (RACE). We established that *MsNAC022* transcripts are cleaved at the site complementary to msi-miR164g between nucleotides 10 and 11 from the 5′ end of the miRNA (Fig. 2C). Consistently, *MsNAC022* transcript levels exhibited a pattern opposite to that of *msi-miR164g* in response to drought. This feature of *MsNAC022* expression was more apparent in leaves, as evidenced by induction after 2 h, followed by repression after 4 h and second rising wave until 24 h (Fig. 2D). Collectively, these findings support the notion that msi-miR164g directly cleaves *MsNAC022* transcripts to decrease their abundance in response to drought.

Bioinformatics analysis indicated that MsNAC022 carries a NO APICAL MERISTEM (NAM) domain, a canonical domain of the NAC family of transcription factors (Fig. 2E). We determined the transcriptional activity of MsNAC022 using a yeast expression system by separately fusing MsNAC022 and MsDREB6.2 (as positive control) to the DNA binding domain of LexA [42]. Both LexA-MsDREB6.2 and LexA-MsNAC022 activated the transcription of the *LacZ* reporter gene, indicating that MsNAC022 is a transcriptional activator (Fig 2F). As might be expected for a transcription factor, we colocalized MsNAC022-GFP fusion protein with the nuclear localization transcription factor MsDREB6.2-RFP fusion protein to the nucleus of *N. benthamiana* leaf epidermal cells (Fig. S6, see online supplementary material).

**Figure 3:**
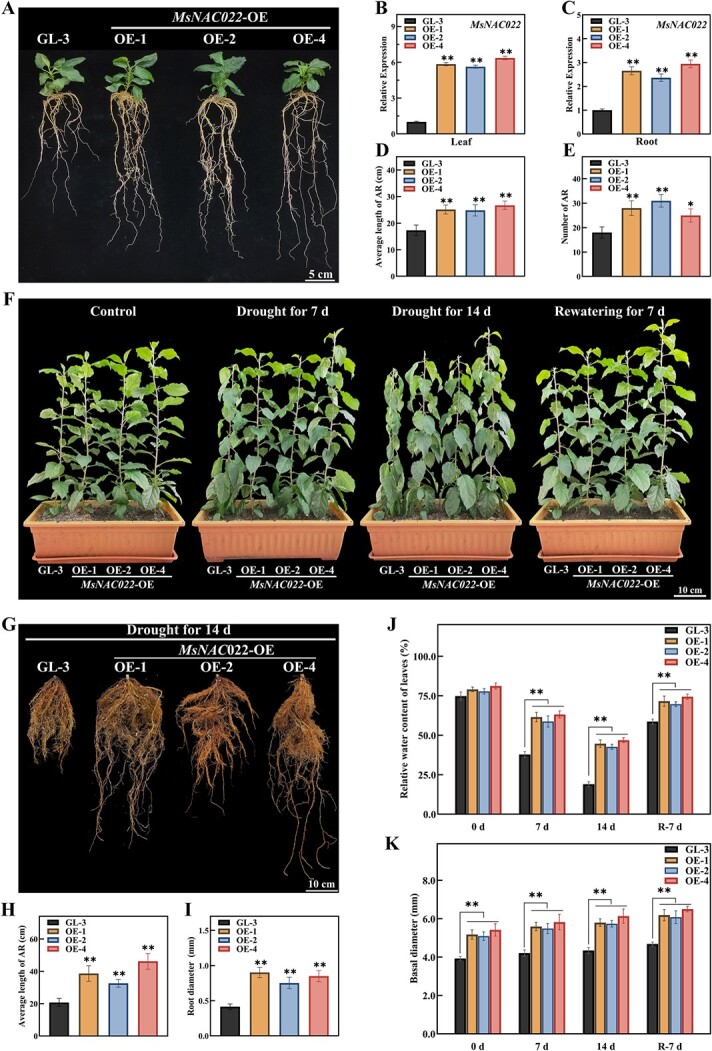
Overexpression of miR164g-resistant *MsNAC022* enhances drought tolerance of transgenic apple plants. **A** Phenotypes of 60-days-old non-transgenic ‘GL-3’ and *MsNAC022-*OE apple plants. Scale bar, 5 cm. **B**, **C***MsNAC022* transcript levels in wild-type and transgenic apple plants. **D**, **E** Average length and number of adventitious roots in the indicated genotypes. **F** Enhanced drought tolerance in *MsNAC022*-OE apple plants compared to nontransgenic ‘GL-3’ plants. The size of the pot: 48 cm × 20 cm × 13 cm. Scale bar, 10 cm. **G** Root system of nontransgenic ‘GL-3’ and transgenic*MsNAC022*-OE apple lines after 14 d drought treatment. Scale bar, 10 cm. **H**, **I** Average length and diameter of adventitious roots in nontransgenic ‘GL-3’ plants and *MsNAC022*-OE apple lines after 14 d drought treatment. **J**, **K** Relative water content in leaves and basal diameter from nontransgenic and *MsNAC022*-OE apple plants after 14 d drought stress and 7 d rewatering (R-7 d). Data are shown as means ± SD from three biological replicates. Asterisks indicate significant differences between the transgenic lines and ‘GL-3’ plants (^*^P < 0.05, ^**^P< 0.01, based on Duncan’s multiple range test).

### Heterologous expression of the *msi-miR164g*-*MsNAC022* module in Arabidopsis alters plant resistance to drought, osmotic and salt stresses

To investigate the potential function of the *msi-miR164g-MsNAC022* module*,* we individually overexpressed *msi-miR164g* and *MsNAC022* in Arabidopsis. We isolated three independent homozygous T_3_ transgenic lines for each construct with high expression levels for analysis, which *msi-miR164g* transcripts were increased by 27–30 and 14–16 times in transgenic lines leaves and roots, respectively. As for *MsNAC022* transcripts, the expression levels were increased by 29–32 and 17–19 times in transgenic lines leaves and roots, respectively (Fig. S7A–E, see online supplementary material). Consistent with the published phenotypes associated with overexpression of its Arabidopsis counterparts as positive regulators of root development, *MsNAC022* overexpression (OE) resulted in longer and more elaborate root systems (Fig. S8C, D, see online supplementary material). By contrast, *msi-miR164*g OE plants exhibited shorter primary roots compared to wild type (Fig. S8A, B, see online supplementary material). To further investigate the drought response of *msi-miR164*g-OE and *MsNAC022*-OE plants, we conducted drought survival assays. After long-term water deprivation, *msi-miR164*g-OE plants exhibited significantly lower survival rates than the wild type (*P* < 0.05; 56% in wild type; 27%,40%, and 33% in the three *msi-miR164*g-OE lines), while *MsNAC022*-OE plants fared better under the same conditions (*P* < 0.05; 45% in wild type; 65%, 64%, and 61% in the three *MsNAC022*-OE lines) (Fig. S8E–H, see online supplementary material). In addition, transcript abundance for four miR164-targeted *AtNAC*s, especially Arabidopsis *NAC022*, was much lower in the three *msi-miR164g*-OE lines compared to the nontransgenic wild-type control, confirming the targeting of NAC022 transcripts by miR164g (Fig. S8I–L, see online supplementary material). Moreover, as drought and salinity both trigger osmotic stress, we examined the phenotypes of *msi-miR164g*-OE and *MsNAC022*-OE lines in response to mannitol osmotic and salt treatment. *Msi-miR164g*-OE lines were more sensitive to osmotic and salt stress, while *MsNAC022*-OE plants showed greater tolerance against these stresses (Fig. S9A, B, see online supplementary material). Collectively, these results suggest that plant resistance to drought, osmotic and salt stresses are negatively and positively modulated by msi-miR164g and MsNAC022, respectively.

### Overexpression of miR164g-targeted *MsNAC022* enhances drought tolerance in transgenic apple plants

To substantiate the function of the msi-miR164g-*MsNAC022* module in *M. sieversii*, we used Agrobacterium (*Agrobacterium tumefaciens*)-mediated transformation of the apple cultivar ‘GL-3’ to generate transgenic plants overexpressing miR164g-resistant forms of *MsNAC022*. We selected three independent transgenic lines (*MsNAC022-*OE-1, OE-2, OE-4) with high *MsNAC022* transcript levels for functional studies (Fig. S7F, G, see online supplementary material). The *MsNAC022* transcripts were increased by 5.62–6.35 and 2.36–2.94 times in transgenic lines leaves and roots, respectively (Fig. 3A–C). Phenotypic analysis showed that as in the Arabidopsis assays (Fig. S8C, D, see online supplementary material), overexpression of *MsNAC022* also resulted in an enhanced roots system in apple, with both longer and more numerous adventitious roots (AR) compared to the non-transgenic ‘GL-3’ apple plants (Fig. 3D, E).

To investigate the role of MsNAC022 on drought stress, we performed a 14-d drought treatment on soil-grown nontransgenic ‘GL-3’ and *MsNAC022-*OE plants. The decrease in soil moisture caused more leaf curling and wilting in ‘GL-3’ plants than in *MsNAC022*-OE lines after 7 d and 14 d of drought treatment (Fig. 3F; Fig. S10A, see online supplementary material). In addition, *MsNAC022*-OE lines recovered from severe drought stress more completely than ‘GL-3’ plants after 7 d rewatering (Fig. 3F). Moreover, *MsNAC022*-OE lines formed vigorous roots systems and exhibited higher relative water content in their leaves relative to nontransgenic control plants (Fig. 3G–J). *MsNAC022*-OE plants also showed greater tolerance to salinity and mannitol osmotic treatment (Fig. S11A, see online supplementary material), which was in line with the phenotypes observed upon *MsNAC022* overexpression in Arabidopsis.

**Figure 4:**
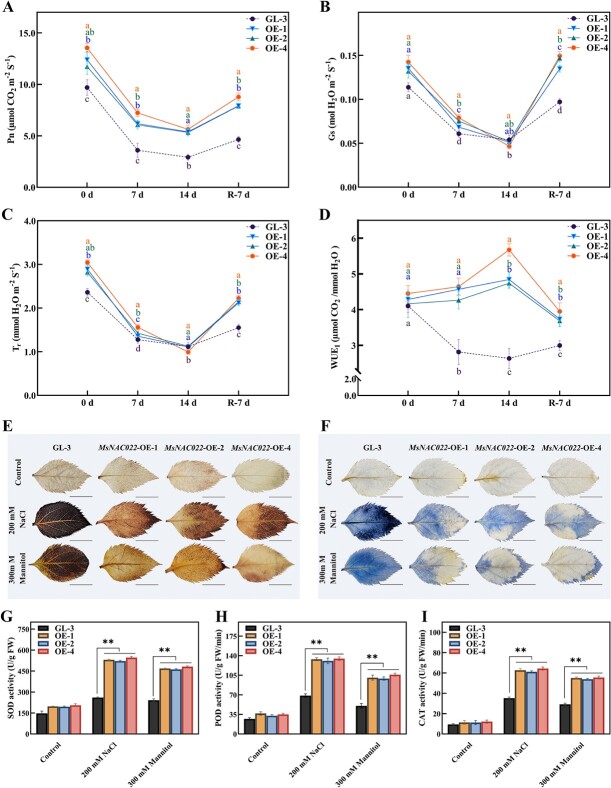
Changes in photosynthetic parameters and ROS scavenging systems of nontransgenic and *MsNAC022* transgenic apple plants under abiotic stress. **A**–**D***P*_n_, *G*_s_, *T*_r_, and *WUE*_I_ of nontransgenic and *MsNAC022* transgenic apple plants after 14 d drought stress and R-7 d. Data are means ± SD from five biological replicates. Different letters indicate significant differences between the transgenic lines and ‘GL-3’ plants (*P* < 0.05, based on Duncan’s multiple range test). **E**, **F** DAB (**E**) and NBT (**F**) staining of nontransgenic and *MsNAC022* transgenic apple leaves under salt and osmotic stress. Scale bars, 1 cm. **G**–**I** SOD (**G**), POD (**H**), and CAT (**I**) activity in nontransgenic and *MsNAC022* transgenic leaves subjected to salt and osmotic stress. Data are shown as means ± SD from three biological replicates. Asterisks indicate significant differences between the transgenic lines and ‘GL-3’ plants (^**^P < 0.01, based on Duncan’s multiple range test).

Many studies have reported that drought inhibits photosynthesis [43, 44]. To determine whether MsNAC022 might mitigate such adverse effects, we monitored the photosynthetic capacity of *MsNAC022*-OE plants during drought stress. Photosynthetic rate (*P*_n_), stomatal conductance (*G*_s_) and transpiration rate (*T*_r_) all decreased as water deprivation was imposed for up to 14 d, but increased upon rewatering. *P*_n_ in *MsNAC022-*OE apple plants was higher than that in ‘GL-3’ across the entire experiment, while *G*_s_ and *T*_r_ in *MsNAC022-*OE apple plants were higher than those in ‘GL-3’ at the beginning of the experiment (0 d, before drought), reached the same lower levels as ‘GL-3’ plants after 14 d of drought stress, but then also recovered faster than the wild-type plants upon rewatering (Fig. 4A–C). In addition, instantaneous water-use efficiency (*WUE*_I_) which refers to the relationship between plant productivity and water use, was also higher in *MsNAC022*-OE lines compared to ‘GL-3’ under drought stress (Fig. 4D). These results indicate that MsNAC022 enhances drought tolerance by raising both photosynthesis rate and WUE_I_.

Drought and salinity stress are accompanied by excessive ROS that cause oxidative damage [2, 13, 16, 45]. To address the exceptional performance of *MsNAC022*-OE plants in the face of drought stress, we examined the accumulation of H_2_O_2_ using 3,3′-diaminobenzidine (DAB) staining and that of superoxide (O_2_^•–^) with nitro blue tetrazolium (NBT) staining. *MsNAC022*-OE transgenic plants displayed a lighter staining pattern for both DAB and NBT compared to ‘GL-3’, indicating that the activity of ROS scavenging systems is higher in *MsNAC022*-OE plants (Fig. 4E, F). We then turned to a quantification of the underlying enzymes required for ROS scavenging. SOD, POD, and CAT activity levels were all elevated in the leaves of *MsNAC022* overexpression lines compared to nontransgenic ‘GL-3’ plants (Fig. 4G–I). The leaves of *MsNAC022* plants accumulated more proline but exhibited lower levels of malondialdehyde (MDA), a marker of cellular oxidative stress (Fig. S11B, C, see online supplementary material). Together, these results suggest that the increased fitness of *MsNAC022*-OE plants upon drought stress is linked to the higher activity of their ROS scavenging systems.

### MsNAC022 promotes the expression of antioxidant genes

**Figure 5:**
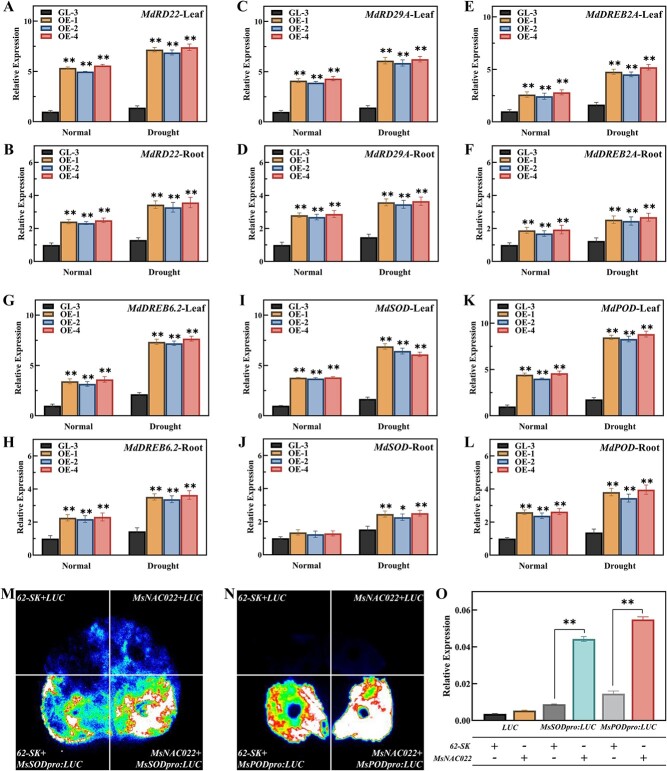
MsNAC022-mediated activation of drought stress-related genes and antioxidant enzyme related genes. **A**–**L**. Expression levels of drought stress-related and ROS scavenging systems related genes in nontransgenic and transgenic apple plants under normal conditions and drought stress. **A**–**B**, *MdRD22*; **C**–**D**, *MdRD29A*; **E**–**F**, *MdDREB2A*; **G**–**H**, *MdDREB6.2*; **I**–**J**, *MdSOD*; **K**–**L**, *MdPOD*. Data are shown as means ± SD from three biological replicates. Asterisks indicate significant differences between the transgenic lines and ‘GL-3’ plants (^*^P < 0.05, ^**^P < 0.01, based on Duncan’s multiple range test). **M**–**O**. Qualitative and quantitative evaluation of MsNAC022-mediated activation of the transcription of genes encoding antioxidant enzyme in a dual-luciferase reporter system, using the *MsSOD* and *MsPOD* promoters to drive *LUC* transcription as reporters. Data are shown as means ± SD from three biological replicates. Asterisks indicate significant differences between the test group and the control group (^**^P < 0.01, based on Duncan’s multiple range test).

We wished to test whether the transcript levels of the drought stress-responsive genes and ROS scavengers also changed in *MsNAC022*-OE plants. Expression levels of seven typical drought stress-responsive genes and two DREB transcription factor were higher in *MsNAC022*-OE plants under normal conditions and drought stress. Likewise, the expression levels of six ROS scavenging genes, *MdSOD*, *MdPOD, MdCAT, MdGST* (*GLUTATHIONE S-TRANSFERASE*), *MdGPX*, and *MdAPX,* were both significantly increased in *MsNAC022*-OE apple plants under normal conditions and drought stress (Fig. 5A–L; Fig. S12A-R, see online supplementary material).

Of the six ROS scavenger genes tested here, the expression of *MdPOD* and *MdSOD* displayed the strongest increase in *MsNAC022*-OE apple plants, which prompted us to investigate whether MsNAC022 directly activates their transcription (Fig. 5I–L). We performed a transient expression assay using a dual-luciferase system in *N. benthamiana* leaf cells, in which we placed the LUC reporter gene under the control of the *MsPOD* or *MsSOD* promoters, yielding the reporter constructs *MsPODpro:LUC* and *MsSODpro:LUC*. LUC activity derived from the *MsPODpro:LUC* and *MsSODpro:LUC* reporters increased substantially when co-infiltrated in *N. benthamiana* leaves with the effector construct *35S:MsNAC022*, compared to the control vector (62-SK) (Fig. 5M–O). In addition, we identified several NAC binding sites among the *cis*-elements in the *MsPOD* and *MsSOD* promoters. However, we failed to detect direct binding between MsNAC022 and the *MsPOD* or *MsSOD* promoters in a yeast one-hybrid assay (Fig. S13, see online supplementary material), indicating that other transcription factors might be recruited to connect MsNAC022 to the *MsPOD* and *MsSOD* promoters and activate their transcription.

### Different *POD* expression levels in *M. sieversii* and *M. domestica* were attributed to the specific *P1* region within the *POD* promoter

**Figure 6:**
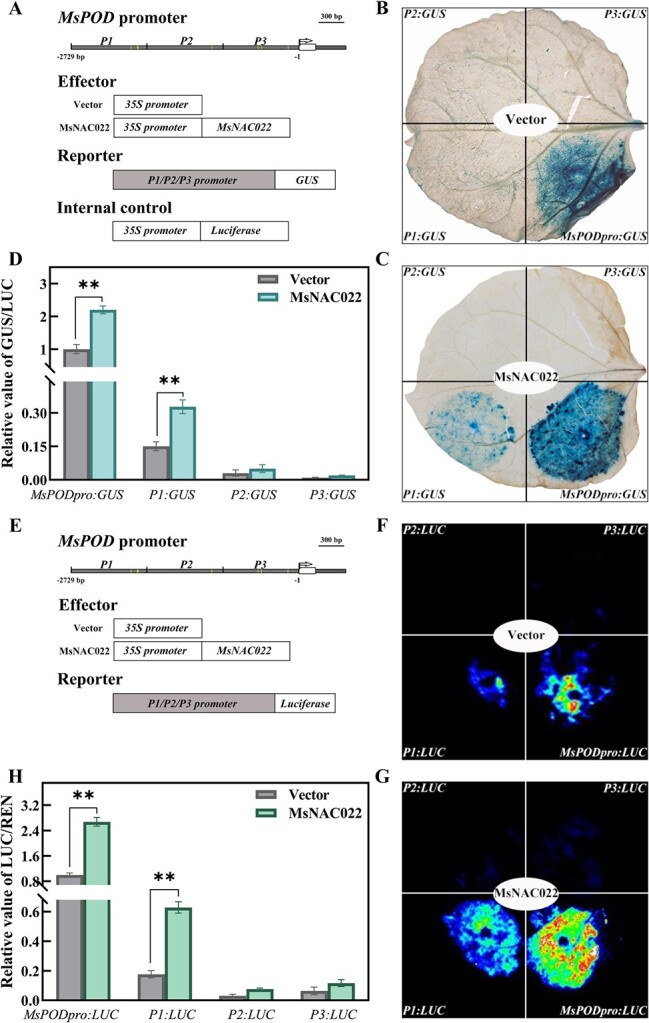
MsNAC022 activates *MsPOD* transcription via the *P1* promoter region. **A** Schematic diagram of the effector and reporter constructs used in the *GUS* reporter transient expression system. **B**–**D***GUS* reporter gene assays of *MsPOD* promoter activity in the presence of MsNAC022 in *N. benthamiana* leaves. **B**, **C** GUS staining of *N. benthamiana* leaves from the transient expression assay. **D** Relative GUS activity (GUS/LUC) from co-infiltrated *N. benthamiana* leaves. **E** Schematic diagram of the effector and reporter constructs used in the *LUC* reporter transient expression system. **F**–**H** Dual-luciferase reporter assay of *MsPOD* promoter activity in the presence of MsNAC022 in *N. benthamiana* leaves. **F**, **G** Images of LUC activity emitted by *N. benthamiana* leaves. **H** Relative LUC activity (LUC/REN) from co-infiltrated *N. benthamiana* leaves. Data are shown as means ± SD from three biological replicates. Asterisks indicate significant differences between treatments and controls (^**^P < 0.01, based on Duncan’s multiple range test).

To further dissect the function of MsNAC022, we selected the *MsPOD* promoter for detailed analysis. We divided the 2.7-kb *MsPOD* promoter into three fragments based on the distribution of predicted NAC binding motifs to drive the transcription of the *β-GLUCURONIDASE* (*GUS*) reporter gene. We then co-infiltrated *N. benthamiana* leaves with the *35S:MsNAC022* effector and each *MsPOD:GUS* construct (Fig. 6A). Staining and relative GUS activity analysis showed that MsNAC022 activates transcription from the *P1* promoter fragment, but not from *P2* or *P3* (Fig. 6B–D). We confirmed these results in a dual-luciferase assay with each *MsPOD* promoter fragment driving the transcription of *LUC* (Fig. 6E–H).

**Figure 7:**
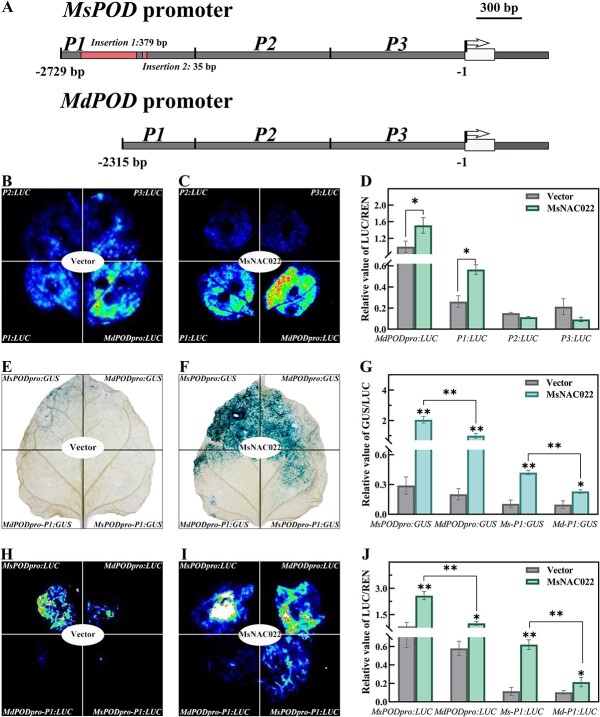
Insertion polymorphisms in the *P1* region of the *MsPOD* promoter enhance transcriptional response in *Malus sieversii*. **A** Schematic diagram of the two *M. sieversii*-specific insertions in the *P1* region of the *MsPOD* promoter. **B**–**D** Dual-luciferase reporter assay of *MdPOD* promoter activity in the presence of MsNAC022 in *N. benthamiana* leaves. **B**, **C** Images of LUC activity emitted by *N. benthamiana* leaves from the transient expression assay. **D** Relative LUC activity (LUC/REN) from co-infiltrated *N. benthamiana* leaves. **E**–**G***GUS* reporter gene assay using the full promoter or the *P1* region of the *POD* promoter from *M. sieversii* and *Malus domestica* in the presence of MsNAC022 in *N. benthamiana* leaves. **E**, **F** GUS staining of *N. benthamiana* leaves from the transient expression assay. **G** Relative GUS activity (GUS/LUC) from co-infiltrated *N. benthamiana* leaves. **H**–**J**. Dual-luciferase reporter assay in the full-length and *P1* region of *POD* promoter activity between *M. sieversii* and *M. domestica* modulated by MsNAC022 in *N. benthamiana* leaves. **H**, **I** Images of LUC activity emitted by *N. benthamiana* leaves from the transient expression assay. **J** Relative LUC activity (LUC/REN) from co-infiltrated *N. benthamiana* leaves. Data are shown as means ± SD from three biological replicates. Asterisks indicate significant differences between treatments and controls (^*^P < 0.05, ^**^P < 0.01, based on Duncan’s multiple range test).

The *P1* region of the *MsPOD* promoter was 910 bp in length and spanned the region from −1819 bp to −2729 bp relative to the ATG start codon. A comparison of the *P1* regions from *M. sieversii* and *M. domestica* identified insertion polymorphisms within this region. The *MsPOD* promoter harbored two fragments (−2143 bp to −2178 bp and − 2213 bp to −2592 bp) of 35 bp and 379 bp, respectively, that are absent from the *MdPOD* promoter (Fig. 7A; Table S2, see online supplementary material)*.* Both insertions were located within the *P1* fragment. We thus characterized the *MdPOD* promoter by dividing the 2.3-kb promoter into three fragments to drive *LUC* or *GUS* transcription. MsNAC022 activated the transcription of both reporter genes from the *P1* region only, as with the *MsPOD* promoter (Fig. 7B–D). We also compared the transcriptional output of the *MsPOD* and *MdPOD* promoters when driving *GUS* or *LUC*. The *MsPOD:GUS* construct resulted in a higher GUS activity than did *MdPOD:GUS* when co-infiltrated with the *35S:MsNAC022* effector, suggesting that the transcriptional activation of *MsPOD* by MsNAC022 is greater than that of *MdPOD*. We noticed that the *P1* region from *M. sieversii* (*Ms-P1:GUS*) resulted in transcription levels about two-fold higher than the *P1* region from *M. domestica* (*Md-P1:GUS*), which explaining the difference in promoter strength between the two apple varieties (Fig. 7E–G). We obtained similar results in a dual-luciferase assay (Fig. 7H–J). Based on these results, the specific *P1* region within the *MsPOD* promoter was attributed to enhanced transcriptional activation of *MsPOD* by MsNAC022, leading to higher accumulation of ROS scavenging systems that partially contribute to the higher drought tolerance observed in *M. sieversii.*

**Figure 8:**
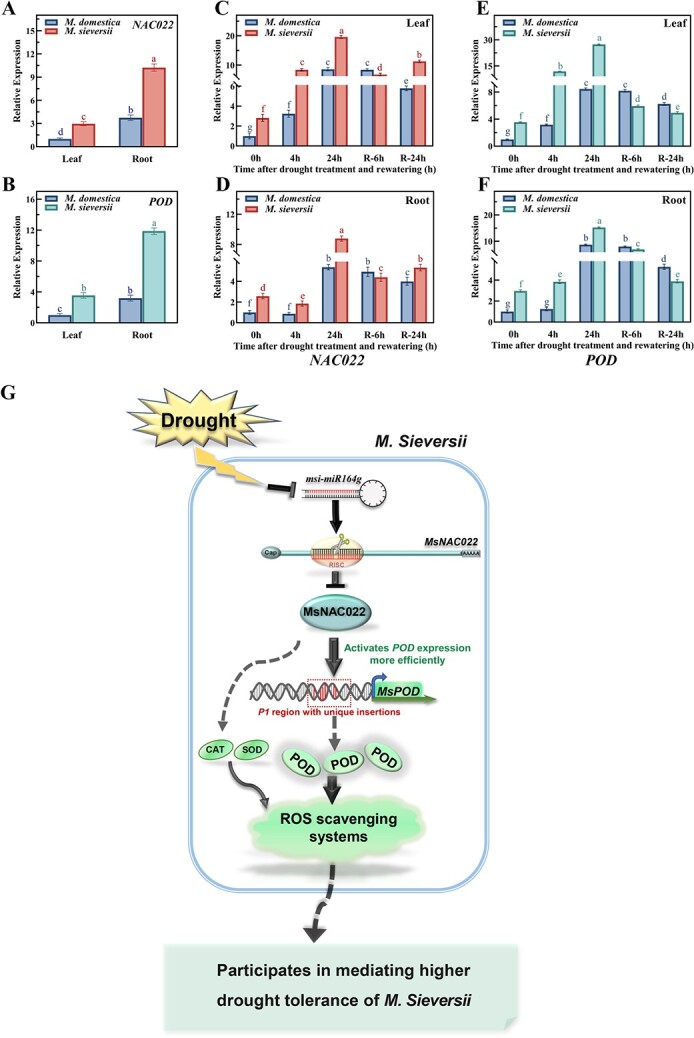
Expression pattern of *NAC022* and *POD* in *Malus sieversii* and *Malus domestica* under normal conditions and short-term osmotic stress and recovery. **A**–**B** Tissue expression levels of *NAC022* and *POD* in *M. sieversii* and *M. domestica* under normal conditions*.***C**–**F** Expression levels of *NAC022* (**C**, **D**) and *POD* (**E**, **F**) in *M. sieversii* and *M. domestica* treated with 30% (w/v) PEG 6000 followed by rewatering (R). Data are shown as means ± SD from three biological replicates. Different letters indicate significant differences (*P* < 0.05, based on Duncan’s multiple range test). **G** Putative model for the miR164g-*MsNAC022* module mediating the transcriptional regulation of ROS scavenger systems contributing to the enhanced drought tolerance of *M. sieversii*: Under drought stress, a drop in miR164g diminishes cleavage of the downstream *MsNAC022* transcripts, whose encoding protein in turn activates the transcription of the downstream gene *MsPOD* and other ROS scavenger genes. As *MsPOD* and *MdPOD* are differentially activated by MsNAC022, enhanced ROS scavenging systems partially contribute to the higher drought resistance of *M. sieversii*.

In light of the different promoter strengths seen for the *M. sieversii* and *M. domestica POD* promoters in transient assays, we measured the transcript levels of *NAC022* and *POD* in the two varieties, under drought stress and recovery conditions. When grown under normal conditions, *NAC022* and *POD* expression was higher in roots relative to leaves, similar to *miR164g* (Fig. 8A, B; Fig. 1C). *NAC022* and *POD* expression levels were higher in *M. sieversii* compared to *M. domestica*. Upon drought stress, *NAC022* and *POD* transcript levels increased rapidly in *M. sieversii* and *M. domestica,* with *M. sieversii* displaying a more pronounced rise. Moreover, *NAC022* and *POD* expression declined to a greater extent in *M. sieversii* after 6 h recovery than in *M. domestica*, indicating a more flexible and plastic response to changes in moisture conditions (Fig. 8C–F). Collectively, these results indicate that the *NAC022-POD* module may be partiallly responsible for the different drought response of *M. sieversii* and *M. domestica.*

## Discussion

### Overexpression of *msi-miR164g* and its target transcript *MsNAC022* alters the drought tolerance of transgenic Arabidopsis and apple plants

Apples are one of the most valuable fruit crops whose productivity and growth is severely restricted by adverse environmental conditions, of which drought is one of the most severe encountered by apples in arid and semi-arid growing areas [3, 4, 46]. Therefore, it is of great scientific and breeding value to identify drought-related components and their underlying molecular mechanisms in apples. miRNAs and their respective targets have roles in responding to various stresses [24, 25, 47–50]. Among these, miR164 is a versatile miRNA with a vital role in plant growth and development. miR164 mediates responses to various abiotic stresses such as salinity, osmosis and drought [26–31, 36, 37, 51–53]. However, the molecular mechanisms by which miR164 and its targets regulate abiotic stress, particularly drought response, are still poorly understood in apples.

Our study characterized the novel miRNA locus *msi-miR164g*, which was differentially expressed under short-term drought stress treatments in *M. sieversii* (Fig. 1D and E). Complementary sequence analysis and cleavage assays determined that *msi-miR164g* directly regulates *MsNAC022* transcript levels in *M. sieversii* (Fig. 2A–C). Overexpression of *msi-miR164g* or *MsNAC022* in Arabidopsis altered tolerance to drought stress. In contrast to the negative regulation of drought stress by *msi-miR164g*, overexpression of *MsNAC022* conferred enhanced drought tolerance in transgenic Arabidopsis and apple (Fig. S8E and F, see online supplementary material; Fig. 3F). Several antioxidant enzymes required for ROS scavenging exhibited high activity levels in *MsNAC022*-OE apple lines, and their encoding genes were highly expressed (Fig. 4G–I; Fig. 5I–L; Fig. S12K–L, see online supplementary material). In particular, we showed here that MsNAC022 activates *MsPOD* transcription (Fig. 5N and O). *POD* promoter analysis in *M. sieversii* and *M. domestica* revealed the presence of specific insertions in *M. sieversii* that contribute to the greater activation of *POD* transcription by MsNAC022 in this variety (Fig. 7). Together, these results suggest that, in response to drought stress, elevated *MsNAC022* transcript levels lead to an activation of *MsPOD* transcription to detoxify the accumulated ROS and thus enhance drought tolerance.

We observed that *msi-miR164g* among all *msi-miR164*s most prominently responds to osmotic treatment. Furthermore, expression analysis of all *msi-miR164*s revealed that *msi-miR164g* has a higher basal expression level than other *msi-miR164* family members under either normal conditions or stress treatment (Fig. 1C–E). We also noted that mature msi-miR164g is highly similar to the recognition sites of its target gene (Fig. 1B). Based on these observations, we speculate that miR164g may be the main contributing member of the *M. sieversii* miR164 family. In our drought treatment assay, *msi-miR164g* in root and leaf exhibited the declined expression trend at 2 h, indicating that *msi-miR164g* respond to drought rapidly (Fig. 1D and E). In contrast to its low level in leaf, *msi-miR164g* mainly expressed in roots (Fig. 1C). Considering that root is the first place to perceive drought stress, thus the effect of drought treatment on *msi-miR164g* expression is more pronounced in root at 2 h (Fig. 1D).

miRNAs cleave the transcripts of their target gene. Previous studies have proved that miR164 directly target the type of NAC transcription factors [18, 27, 30, 32, 34, 39]. The NAC transcription factors directly regulate an array of stress-related genes expression to control drought response [16, 18, 52]. In this study, we established that *MsNAC022* transcripts are targeted by msi-miR164g for cleavage, through dual-luciferase-based miRNA sensor assays and degradome sequencing (Fig. 2A–C). In support of this notion, the transcript levels of *MsNAC022* and *msi-miR164g* showed an opposite relationship under drought stress conditions (Fig. 2D). Furthermore, overexpression of *msi-miR164g* and *MsNAC022* resulted in drought-sensitive and drought-tolerant transgenic plants, respectively (Fig. S8E and F, see online supplementary material; Fig. 4F). Thus, we conclude that msi*-*miR164g negatively mediates drought tolerance by downregulating *MsNAC022* transcript levels. Similarly, NAC transcription factors JUB1 and SNAC3 were also reported to improve the drought resistance by enhancing the ROS scavenging systems in tomato and rice, respectively [17, 18]. In addition, the positive regulation on drought tolerance was also identified in maize NAC transcription factors ZmNAC11, which improves water-use efficiency and upregulates the expression of drought-responsive genes [41]. However, the *miR164*-targeted *NAC* genes in rice act negatively on drought tolerance, implying that the function of this pathway in abiotic stress is divergent in different plant species [52].

Plant water uptake is ultimately determined by the size, properties, and distribution of the root system [54, 55]. Thus, water deficit induces changes in root architecture to adapt to the adverse environment. Previous studies have shown that overexpression of miR164-targeted *NAC* transcription factors induces lateral root development in transgenic Arabidopsis [30]. Similar results were reported in maize and soybean (*Glycine max*), as overexpression of *ZmNAC1* or *GmNAC020* resulted in more lateral roots and greater root density [32, 56]. In our study, transgenic Arabidopsis and apple plants overexpressing *MsNAC022* also showed enhanced root system, especially in transgenic apple plants, the number and length of adventitious roots were significantly increased (Fig. S8C and D, see online supplementary material; Fig. 3A, D, and E). After 14 days of drought stress, the root difference of transgenic apple was more significant than that of the control plant, indicating that MsNAC022 significantly affected the root development of the transgenic plant during drought stress, thus conferring the transgenic apple plant excellent adaptability to drought stress (Fig. 3F–J). Similar to our study, *miR167a* expression was significantly reduced in Arabidopsis under high osmotic stress, which subsequently increased the expression level of target genes and promoted lateral root development, while root structure optimization enhanced the tolerance of transgenic plants under osmotic stress [50]. Our results suggest that the msi*-*miR164g*-MsNAC022* module also affects apple root architecture, thereby conferring apple plants’ response to drought, salt, and osmotic stresses. However, the regulatory mechanisms by which msi*-*miR164g*-NAC022*-mediated root traits enhance stress resistance is unknown. Thus, more detailed molecular and biochemical studies are required to substantiate the regulation of root development by MsNAC022 in future studies.

### The msi-miR164g*-MsNAC022* module enhances drought tolerance in transgenic apple plants by increasing POD activity

Although ROS production is critical for growth, signaling, and development, their reactivity is a double-edged sword. Low ROS levels constitute a stress-signaling component that is beneficial for acclimation in response to stress [12, 57]. However, an overaccumulation of ROS becomes extremely deleterious, initiating oxidative stress that results in cellular damage and ultimately cell death. Under drought stress, photosynthetic rates drop, leading to enhanced electron leakage and photorespiration, both producing excess ROS [13]. Thus, steady-state ROS levels must be tightly regulated. To mitigate the damage caused by ROS overproduction, drought stress upregulates ROS scavenging systems, in the form of both enzymatic and non-enzymatic antioxidants [15]. The level of induction of the antioxidant systems is tightly correlated with the degree of drought tolerance exhibited by plants. Modulation of ROS scavenging systems affects plant survival rates under drought stress. For instance, overexpression of *AUTOPHAGY-RELATED 18a* (*MdATG18a*) increases CAT and POD activities, thus enhancing the drought tolerance of transgenic apples [58]. Likewise, in our previous work, overexpression of the miR171i target gene *SCARECROW-LIKE PROTEINS 26.1* (*MsSCL26.1*) in apples markedly induced *MdAPX* and *MONODEHYDROASCORBATE REDUCTASE* (*MsMDHAR*) expression*,* thereby increasing AsA levels and improving drought tolerance in apples [40].

In the current study, we showed that overexpression of *MsNAC022* promotes SOD, POD and CAT activities in transgenic apple lines compared to nontransgenic ‘GL-3’ plants under drought stress (Fig. 4G–I). In agreement with this observation, H_2_O_2_ accumulation was much lower in *MsNAC022*-OE apple leaves compared to the ‘GL-3’ control under abiotic stress, indicating that greater ROS mitigation may contribute to higher drought tolerance (Fig. 4E and F). Furthermore, the genes required for ROS scavenging systems were induced in *MsNAC022*-OE apple plants (Fig. 5I–L; Fig. S12K–R, see online supplementary material). MsNAC022 activated transcription from the *MsSOD* and *MsPOD* promoters (Fig. 5M–O). These results collectively demonstrate that the mi164g*-MsNAC022* module may mediate the drought response of apples by regulating the expression of genes encoding ROS scavenging enzymes. In addition, our failure to observe direct binding of MsNAC022 on the *MsSOD* and *MsPOD* promoters in a yeast one-hybrid assay suggests that MsNAC022 might not act alone and may recruit other transcription factors or proteins to activate transcription. In our research, seven drought stress-related response and two DREBs gene were significantly increased in *MsNAC022*-OE plants relative to nontransgenic apple plants. Upon drought stress, the response levels of seven drought stress-related genes and two DREB genes were more significant imposed compared to normal conditions (Fig. 5E–H; Fig. S12A–J, see online supplementary material). Furthermore, previous studies have reported that NAC transcription factors can form homodimers or heterodimers or interact with DREBs or C-REPEAT/DRE BINDING FACTORs (CBFs) to enhance plant tolerance to drought and cold, respectively [59–63]. Based on the important regulatory function of DREB transcription factors in plant drought stress, we speculate that the DREB transcription factors may be a potential co-factor of MsNAC022 [42]. Hence, elucidating the concrete action of MsNAC022 in regulating the downstream ROS pathway is our next research interest and will be focused on in the near future.

### Two insertions in the *P1* region of the *MsPOD* promoter may contribute to the differences in drought resistance between *M. sieversii* and *M. domestica*

*M. sieversii* is the likely progenitor of modern cultivated apple(*M. domestica* Borkh.) [64]. Among all cultivated rootstocks, *M. sieversii* is one of the most drought-resistant species in China [7–9]. However, some excellent drought response genes and regulatory mechanisms of *M. sieversii* may have become weaker or even eliminated in long-term domestication, resulting in the lowerobserved drought tolerance of *M. domestica.* Understanding the molecular genetic mechanisms governing drought responses in *M. sieversii* will help breeding programs enhance the survival of important commercial apple cultivars during periods of drought. Upon osmotic treatment, *MITOGEN-ACTIVATED PROTEIN KINASE* (*MAPK*) expression is rapidly upregulated, and *M. sieversii* displays the highest *MAPK* expression levels compared to other apple species [65]. Moreover, our previous study reported that MsDREB6.2 regulates cytokinin metabolism and participates in stomatal regulation, root development and aquaporin gene expression, thereby enhancing drought tolerance [42]*.* Based onthese results, the drought response machinery in *M. sieversii* is thought to be more flexible and plastic than that in *M. domestica.* Despite much progress, the molecular mechanisms behind thediscrepancy of drought tolerance between *M. sieversii* and *M. domestica* are not well understood.

In our study, we showed that upon drought stress, ROS overaccumulation slows down in *MsNAC022*-OE plants overexpressing a miR164g-resistant form of the gene (Fig. 4E and F). As one of the main ROS scavengers, *POD* transcription was activated by MsNAC022 specifically through the *P1* region (Figs 5N and O, and 6). Furthermore, sequence comparison of the *P1* region highlighted two insertions present only in *M. sieversii* (Fig. 7A; Table S2, see online supplementary material). We further established that MsNAC022 activates transcription from the *Ms-P1* promoter fragment to greater levels than with *Md-P1*, which lacks these twounique insertions (Fig. 7E–J). In addition, *NAC022* and *POD* expression was more strongly induced in response to changes in moisture conditions in *M. sieversii* than in *M. domestica* (Fig. 8C–F). Based on these findings, we propose a putative model for the transcriptional regulation of ROS scavenger systems that is mediated by the miR164g-*MsNAC022* module, which may contribute to the enhanced drought tolerance of *M. sieversii* (Fig. 8G). Under drought stress, rapidly declining miR164g levels alleviate the cleavage of *MsNAC022* transcripts, whose encoding transcription factor in turn activates the transcription of its downstream gene *MsPOD* and other ROS scavenger genes. As MsNAC022 differentially activates *MsPOD* and *MdPOD*, the enhanced ROS scavenging systems might partially contribute to the higher drought resistance of *M. sieversii*.

Drought stress is becoming one of the most critical determinants that limit apple production. It is highly desirable to breed new apple varieties with high water uptake efficiency or drought tolerance through biotechnology or molecular marker-assisted breeding, but such efforts have met only limited success thus far. *M. sieversii* was widely used because of its excellent drought resistance [7–9]. Dissecting the unique molecularmechanism of drought responses in *M. sieversii* will provide valuable genetic resources for breeding new drought-resistant rootstocks. Our work identified a novel regulatory module acting in *M. sieversii* drought response, which may have applications in apple breeding to increase plant fitness during drought stress through modulating the miR164g*-MsNAC022* circuitry, ultimately mitigating ROS damage.

## Materials and methods

### Plant materials and drought stress treatments

Tissue-cultured plants of *M. sieversii* and *M. domestica* were grown at 23°C and 40% relative humidity under a 16 h/8 h (day/night) photoperiod as described [40]. The apple plant ‘GL-3’ (*M. domestica* Borkh.) from the laboratory of Dr Zhihong Zhang (Liaoning, China) was also cultured under the conditions described above, which will be used for genetic transformation and abiotic stress treatments as previously described [66]. Rooted *M. sieversii* and *M. domestica* plants that were hydroponically precultured in half-strength Hoagland nutrient were subjected to drought stress. The plants were treated as described in previous studies with a solution of 20% (w/v) PEG 6000 or 30% PEG 6000 (Xilong Scientific, Shantou, China) for various periods of time [40, 42]. Samples were then separately harvested at 0, 2, 4, 12, and 24 h after drought stress and 6 and 24 h after rewatering, and were quickly frozen in liquid nitrogen for RNA isolation and expression analysis.

The wild-type and transgenic Arabidopsis used in this study were in the *Columbia-0* ecotype background. After sterilizing, seeds were planted on half-strength MS medium and then stratification at 4°C for 3 d. At 14 d later, seeding was transplanted to soil and grown in the greenhouse at 22°C with 16 h light/8 h dark cycle.

### Cloning of the msi-miR164g and its target gene *MsNAC022*

The msi-miR164g and the cleaving to miR164g-targeted gene *MsNAC022* was screened via analyses of miRNAs databases of high-throughput small-RNA sequencing and degradome sequencing of *M. sieversii* which were obtained in our previous study [40].

To validate the new predicted miRNA, stem-loop reverse transcription PCR were used to obtain the mature of miR164g from *M. sieversii*, then were cloned into the pTOPO-blunt sample vector (Aidlab, Beijing, China) and clones were confirmed through sequencing. The coding sequence of *MsNAC022* was amplified by reverse transcription PCR (RT-PCR) of *M. sieversii* RNA*,* and confirmed through sequencing. Primer sequences were listed in Table S3, see online supplementary material.

### Analysis of *cis*-acting elements related to abiotic stress in *msi-miR164g* promoter of *M. sieversii*

The *M. sieversii* genomic DNA was extracted using a Plant Genomic DNA Kit (Tiangen, Beijing, China). We checked the *msi-miR164g* promoter reference sequences by blast the GDR apple genomic database (https://www.rosaceae.org/) with the msi-miR164g precursor sequences obtained from our miRNAs databases, then we designed a specific primer and cloned the *miR164g* promoter sequences from *M. sieversii.* The msi-miR164g promoter sequences were submitted to PLANTCARE (http://bioinformatics.psb.ugent.be/webtools/plantcare/html/) to perform *cis*-acting elements analysis.

### RNA extraction and analysis of expression levels by quantitative real-time PCR (qRT-PCR)

The Arabidopsis total RNA was extracted with TRIzol Reagent (CWBIO, Beijing, China). Small RNA was extracted using an EASYspin Plant microRNA Extract kit (RN40, Aidlab, Beijing, China). The DNA was removed by treating with RNase-free DNase I (RN34, Aidlab, China). After the detection of RNA samples quality by agarose electrophoresis, the same amount of RNA samples (1 μg) was used to generate cDNA with oligo dT primers and specific stem-loop reverse transcription primers of miRNA164 family members, respectively. The qRT-PCR was performed with SYBR Green Mix (CW0659, CWBIO, China). NCBI primer-blast online tools (https://www.ncbi.nlm.nih.gov/tools/primer-blast/index.cgi?LINK_LOC=BlastHome) and qPrimerDB (https://biodb.swu.edu.cn/qprimerdb/) online database tools were used to aid in the design of specific primers. The reactions were incubated in a Rotor-Gene Q Machine (Qiagen, Hilden, Germany), *AtActin2* and *Histone H3* were used as internal controls for Arabidopsis and apple, respectively. Melting curve assay were performed to detection the specificity of qRT-PCR reactions. Gene amplification efficiencies and relative expression levels were analysed using the previous reported method [67]. All primers were listed in Table S3, see online supplementary material.

### Phylogenetic analysis of miR164s and NAC family members

A total of 126 miR164 precursors from 36 plant species and the NAC transcription factors amino acid sequences from Arabidopsis and apple were used as queries in BLAST searches of the miRBase 22.1, TAIR, and GDR databases, respectively. The phylogenetic analysis was conducted using MEGA X and the neighbor-joining method with 1000 bootstrap replicates [68]. The phylogenetic trees were modified using the Evolview v3 tool [69].

### Dual luciferase-based miRNA sensor assay in *N. benthamiana* leaves

The msi-miR164g potential cleavage sites of MsNAC022 were introduced into the *Avr*II/*Age*I sites of the pGreen-dual-luc-ORF-sensor vector as previously described, also its synonymous mutation sequences which were used for a negative control [70]. The primary of msi-miR164g with 482 bp sequence from *M. sieversii* was driven by the cauliflower mosaic virus 35S (CaMV*35*S) promoter in the pGreenII 0029 62-SK vector. The pGreen-dual-luc-ORF-sensor vector also carries the *REN* gene and was used as a positive control. Transformation and infiltration were performed as described [71].

### Analysis of transcriptional activation activity and subcellular localization for MsNAC022

To investigate the transcriptional activation activity of MsNAC022, the full-length *MsNAC022* sequence lacking the termination codon was introduced into pEG202 vector. Sequencing-confirmed plasmid was transformed into yeast strain EGY48, and MsDREB6.2 was used for positive control [42]. Transcriptional activity assay was performed as described [72].

For the colocalization of MsNAC022, the full-length *MsNAC022* sequence without the termination codon was fusion expressed with GFP by inserting into the *pCAMBIA1302* vector (https://cambia.org/welcome-to-cambialabs/cambialabs-projects/cambialabs-materials-and-methods-developed-in-cambialabs/). The nuclear localization transcription factor MsDREB6.2^[42]^ was fusion expression with RFP by inserted into the *pCAMBIA1300* vector modified by us. Vectors were transformed into *A. tumefaciens* strain GV3101. After *Agrobacterium* microbial concentration reached OD_600_ = 1.0, they were equally mixed and then were transiently transformed in *N. benthamiana* leaves and confocal fluorescence observation was performed as described [73].

### Construction of overexpression vectors and transgenic plants

To generate the *msi-miR164g* overexpression vector, the 482 bp sequence from *M. sieversii* containing the miR164g stem-loop structure was cloned and inserted into the downstream of the CaMV*35*S promoter in plant transformation vector *pCAMBIA1301*. Similarly, the coding sequence of *MsNAC022* with synonymous mutations in the miR164g cleavage sites was amplified using overlapping PCR and fused into *pCAMBIA1301* vector to construct the *MsNAC022* overexpression vector. *A. tumefaciens* strain EHA105 transformed with these vectors was used for genetic transformation in Arabidopsis and apple ‘GL-3’ plants as previously described [74, 75]. After Hygromycin B selection, we performed PCR analysis to identify the presence of the transgenes and the Hygromycin coding sequences in putative transgenic lines; total RNA was also isolated to check the overexpression of *msi-miR164g* and *MsNAC022* by qRT-PCR. In addition, apple leaves GUS staining assay was performed to identification the reporter gene of *MsNAC022-*OE transgenic ‘GL-3’ apple lines by a modified method [40, 76].

### Drought stress treatments, salinity, and osmotic treatment for transgenic plants

For drought stress treatment, the wild type plants and the overexpression T_3_ Arabidopsis lines were transplanted to the same pot (48 cm × 20 cm × 13 cm) and grew for 2.5 weeks, then were grown under drought stress for 2 weeks or 3 weeks (water was withheld); 5-month-old nontransgenic ‘GL-3’ apple plants and transgenic plants were also transplanted to the same pot and performed 2 weeks drought stress treatment (water was withheld).

For salinity and osmotic treatment, 150 mM NaCl and 250–300 mM mannitol were added into the half-strength MS medium to treat the 14-d wild-type plants and the overexpression T_3_ Arabidopsis lines as described [40, 77]; 200 mM NaCl and 300 mM mannitol were also added into the MS subculture medium to treat nontransgenic ‘GL-3’ apple plants and transgenic plants as described [40, 66].

### Determination of photosynthetic parameters and leaves relative water content

During drought stress, the photosynthetic capacity of *MsNAC022*-OE plant was monitored on sunny days between 9 and 11 a.m. with a photosynthetic apparatus (Li-6400; LICOR, Huntington Beach, CA, USA). Five measurements were performed for each group as previously described [58]. The relative water content of leaves was determined as described [3].

### Determination of several ROS physiological indicators and staining of DAB, NBT

The measurement of ROS physiological indicators, SOD, POD, and CAT activity, Proline and MDA levels in leaves was performed as previously described [3, 78]. DAB and NBT staining using a modified method as described [79].

### Analysis of downstream genes expression and dual-luciferase assay

Expression levels of typical stress-responsive genes *RD22*, *RD29A*, *RD29B, RD26, ERD1, ERD10*, and *LEA7* were examined in transgenic apple plants by qRT-PCR, as well as the various ROS scavenging systems related genes which were identified in the previous research, such as *MdPOD, MdSOD, MdCAT, MdGST, MdGPX*, and *MdAPX* [40, 80–83]. We also selected two DREB transcription factors MdDERB2A and MdDERB6.2 based on their essential roles in drought stress response [42, 84].

The genomic DNA was extracted from *M. sieversii* and *M. domestica* using a Plant Genomic DNA Kit (TIANGEN, China). Then promoters of *MsPOD, MdPOD*, and *MsSOD* were cloned and fused to pGreenII 0800-LUC vector, respectively. The full-length CDS of *MsNAC022* was driven by the CaMV35S promoter in the pGreenII 0029 62-SK vector, then corresponding vectors were transformed into *A. tumefaciens* strain GV3101 harboring the pSoup plasmids. *LUC* promoter activity analysis was examined as described [85], as well as the *LUC* activities of the full-length and different fragments of *MsPOD* and *MdPOD* promoters.

### *GUS* reporter assay in *N. benthamiana* leaves

The promoter of *MsPOD and MdPOD* was divided into three fragments according to the distribution of NAC binding element, the full-length promoters and different fragments were inserted into the *pCAMBIA1301* vector, which contains the *GUS* reporter gene vector of *35S:LUC* that was used for internal control. Vectors were transformed into *A. tumefaciens* strain GV3101. After *Agrobacterium* concentration was shaken and adjusted to OD_600_ = 1.0, various Agrobacterium that harbour effector, reporter, and internal controls were equally mixed and left to stand in the dark for about 1 h before infiltration. Transient transformation in *N. benthamiana* leaves was performed as described [70]. The histochemical staining of *GUS* and *GUS* activity analysis were performed as described [76].

### Statistical analyses

All statistical analyses were performed via the one-way ANOVA followed by Duncan’s multiple range test, using the SPSS22.0 for Windows (SPSS Inc., Chicago, IL, USA). Three independent biological replicates were performed for each determination. Data are shown as mean ± standard deviation (SD), differences asterisks between these results were considered as statistically significant (^*^P < 0.05, ^**^P < 0.01).

### Accession numbers

Sequence data from this article can be found in the TAIR databse (https://www.arabidopsis.org) and the Genome Database for Rosaceae website (https://www.rosaceae.org/): MsNAC022 (MD10G1198400); MsDREB6.2 (MD15G1365500); MdRD22 (MD15G1098800); MdRD29A (MD01G1201000); MdRD29B (MD07G1268800); MdRD26 (MD03G1222700); MdERD1 (MD06G1128400); MdERD10 (MD15G1003900); MdLEA7 (MD03G101800); MdDREB2A (MD01G1158600); MdDREB6.2 (MD15G1365500); MdPOD (MD00G1112500); MdSOD (MD00G1051500); MdCAT (MD06G1008600); MdGPX (MD06G1081300); MdGST (MD04G1111600); MdAPX (MD08G1150400); AtNAC022 (AT1G56010); AtNAC1L (AT3G12977); AtNAC080 (AT5G07680); AtNAC100 (AT5G61430).

## Acknowledgments

We thank Prof. Zhihong Zhang from Shenyang Agricultural University for providing the ‘GL-3’ apple plants. This work was supported by the National Key R&D Program of China (Grant No. 2019YFD1000102–02), the National Natural Science Foundation of China (Grant No. 31972390), the Construction of Beijing Science and Technology Innovation and Service Capacity in Top Subjects (Grant No.CEFF–PXM2019_014207_000032), and the 2115 Talent Development Program of China Agricultural University.

## Author contributions

X.P., Y.L., and T.-H.L. conceptualized this project and designed all experiments. Y.-T.W., X.P., and C.F. treated plant materials and performed the RNA extraction and gene expression analysis. X.P., C.F., and X.Zha performed the dual luciferase and GUS reporter assay. Y.-Y.W. and X.Zha contributed to the phylogenetic analysis of miR164 and NAC family members. Y.-Y.W. and Y.-T.S. contributed to the transcriptional activity analysis and subcellular localization of MsNAC022. Y.-Q.X., Z.-F.Z., and X.Zho. measured the physiological indicators. C.F. monitored the photosynthetic characteristics of *MsNAC022-*OE plants during drought stress. B.-Y.D. and C.W. contributed to the plasmid construction and Arabidopsis and apple genetic transformation. X.P., Y.L. and T.-H.L. wrote and revised the manuscript. All authors have read and agreed to the published version of the manuscript.

## Data availability

The data that support the findings of this study are available from the corresponding author upon reasonable request.

## Conflict of interest

The authors declare no competing interests.

## Supplementary data

Supplementary data is available at *Horticulture Research* online.

## Supplementary Material

Web_Material_uhac192
