# Supplementary material for: miR164g-MsNAC022 acts as a novel module mediating drought response by transcriptional regulation of reactive oxygen species scavenging systems in apple
Source: Hortic Res. 2022 Aug 30;9:uhac192. doi: 10.1093/hr/uhac192 (PMC9630969; doi:10.1093/hr/uhac192)
Supplement: Web_Material_uhac192 [file web_material_uhac192.pdf]

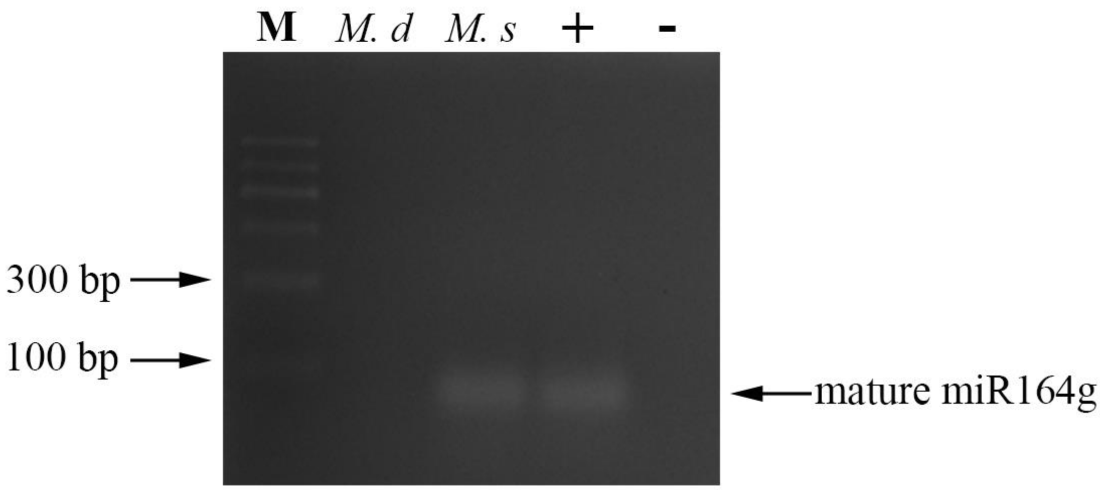

Fig. S1 Validation of the new predicted miRNc11 in *M. domestica* and *M. sieversii*.
M. d: *M. domestica*; *M. s*: *M. sieversii*; M: Marker II; + : positive control of stem-loop reverse transcription products; -: negative control.

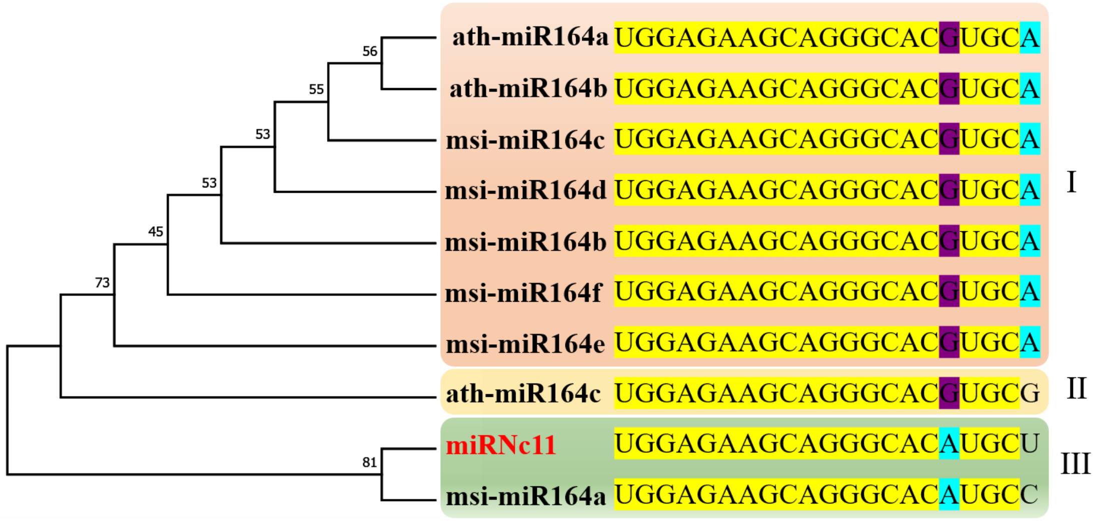

Fig. S2 Multiple sequence analysis of msi-miR164 family members.

Multiple sequence alignment of miR164 family members from *M. sieversii* and Arabidopsis, miRNc11 is marked with red font.

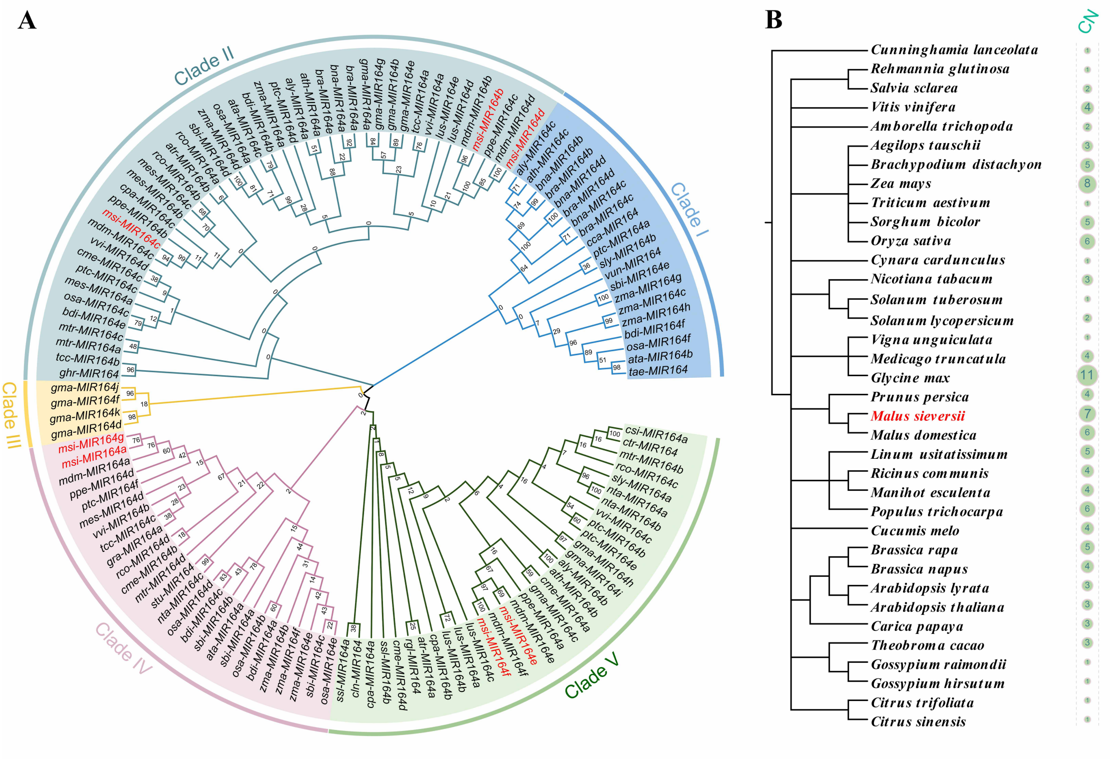

Fig. S3 Phylogenetic analysis of 126 miR164 family members in 36 plant species.

A. Phylogenetic analysis of 126 miR164 family members in 36 plant species, members of the msi-miR164 family are marked with red font. **B.** Statistics of miR164 family numbers in 36 plant species. *M. sieversii* is marked with red font. CN, copy numbers for 164 family members.

A

miRNA Acc.	Target Acc.	Expect	UPE	Alignment	Target Description
msi-miR164g	MD10G1198400	1.0	N/A	miRNA 21 UCGUACACGGGACGAAGAGGU 1<br>:::: ::::::::::::::::<br>Target 669 AGCAAGUGCCCUGCUUCUCCA 689	pacid=40090013 locus=MD10G1198400 annot-version=v1.1

B

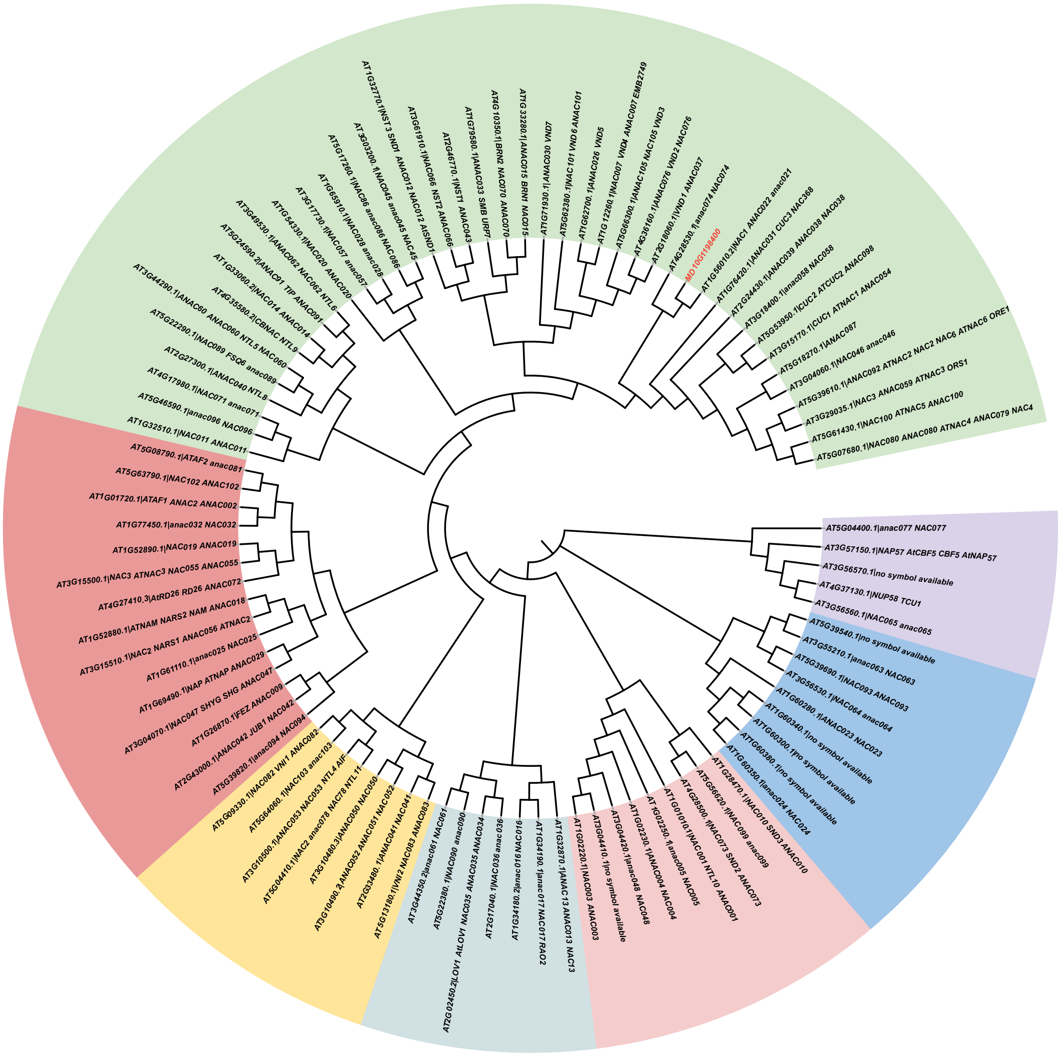

Fig. S4 Target prediction of msi-miR164g in apple and Phylogenetic analysis of msi-miR164g target gene.

A. Target prediction of msi-miR164 in the apple transcript database (https://www.zhaolab.org/psRNATarget/). **B**. Phylogenetic analysis of msi-miR164g-targeted MD10G1198400 and NAC transcription factors from *Arabidopsis thaliana* (Arabidopsis).

A

miRNA Acc.	Target Acc.	Expect	UPE	Alignment	Target Description	Inhibition	Multiplicity
ath-miR164	AT5G07680.2	0.5	N/A	miRNA 21 ACGUGCACGGGACGAAGAGGU 1<br>: .::::::::::::::::::<br>Target 651 UUUACGUGCCCUGCUUCUCCA 671	| Symbols: ANAC080, NAC080 | NAC domain containing protein 80 | chr5:2435983-2437499 FORWARD LENGTH=1192	Cleavage	1
ath-miR164	AT5G61430.1	0.5	N/A	miRNA 21 ACGUGCACGGGACGAAGAGGU 1<br>: .::::::::::::::::::<br>Target 840 UCUACGUGCCCUGCUUCUCCA 860	| Symbols: ANAC100, ATNAC5, NAC100 | NAC domain containing protein 100 | chr5:24701122-24702761 REVERSE LENGTH=1425	Cleavage	1
ath-miR164	AT1G56010.2	1.0	N/A	miRNA 21 ACGUGCACGGGACGAAGAGGU 1<br>:::::: :::::::::::::<br>Target 802 AGCACGUACCCUGCUUCUCCA 822	| Symbols: NAC1, ANAC022 | NAC domain containing protein 1 | chr1:20946571-20949281 REVERSE LENGTH=1393	Cleavage	1
ath-miR164	AT3G12977.1	1.5	N/A	miRNA 21 ACGUGCACGGGACGAAGAGGU 1<br>:::.:: :::::::::::::<br>Target 717 AGCAUGUACCCUGCUUCUCCA 737	| Symbols: | NAC (No Apical Meristem) domain transcriptional regulator superfamily protein | chr3:4143726-4145967 FORWARD LENGTH=1053	Cleavage	1

B

miRNA Acc.	Target Acc.	Expect	UPE	Alignment	Target Description	Inhibition	Multiplicity
msi-miR164g	AT3G12977.1	1.0	N/A	miRNA 21 UCGUACACGGGACGAAGAGGU 1<br>::::::: :::::::::::::<br>Target 717 AGCAUGUACCCUGCUUCUCCA 737	| Symbols: | NAC (No Apical Meristem) domain transcriptional regulator superfamily protein | chr3:4143726-4145967 FORWARD LENGTH=1053	Cleavage	1
msi-miR164g	AT5G07680.2	1.5	N/A	miRNA 21 UCGUACACGGGACGAAGAGGU 1<br>.: ::::::::::::::::<br>Target 651 UUUACGUGCCCUGCUUCUCCA 671	| Symbols: ANAC080, NAC080 | NAC domain containing protein 80 | chr5:2435983-2437499 FORWARD LENGTH=1192	Cleavage	1
msi-miR164g	AT5G61430.1	1.5	N/A	miRNA 21 UCGUACACGGGACGAAGAGGU 1<br>.: ::::::::::::::::<br>Target 840 UCUACGUGCCCUGCUUCUCCA 860	| Symbols: ANAC100, ATNAC5, NAC100 | NAC domain containing protein 100 | chr5:24701122-24702761 REVERSE LENGTH=1425	Cleavage	1
msi-miR164g	AT1G56010.2	2.0	N/A	miRNA 21 UCGUACACGGGACGAAGAGGU 1<br>:::: :: :::::::::::::<br>Target 802 AGCACGUACCCUGCUUCUCCA 822	| Symbols: NAC1, ANAC022 | NAC domain containing protein 1 | chr1:20946571-20949281 REVERSE LENGTH=1393	Cleavage	1

Fig. S5 Candidate target genes prediction of ath-miR164 and msi-miR164g in the Arabidopsis transcript database.

A. Target prediction of ath-miR164 in the Arabidopsis transcript database (https://www.zhaolab.org/psRNATarget/). **B.** Target prediction of msi-miR164g in the Arabidopsis transcript database (https://www.zhaolab.org/psRNATarget/).

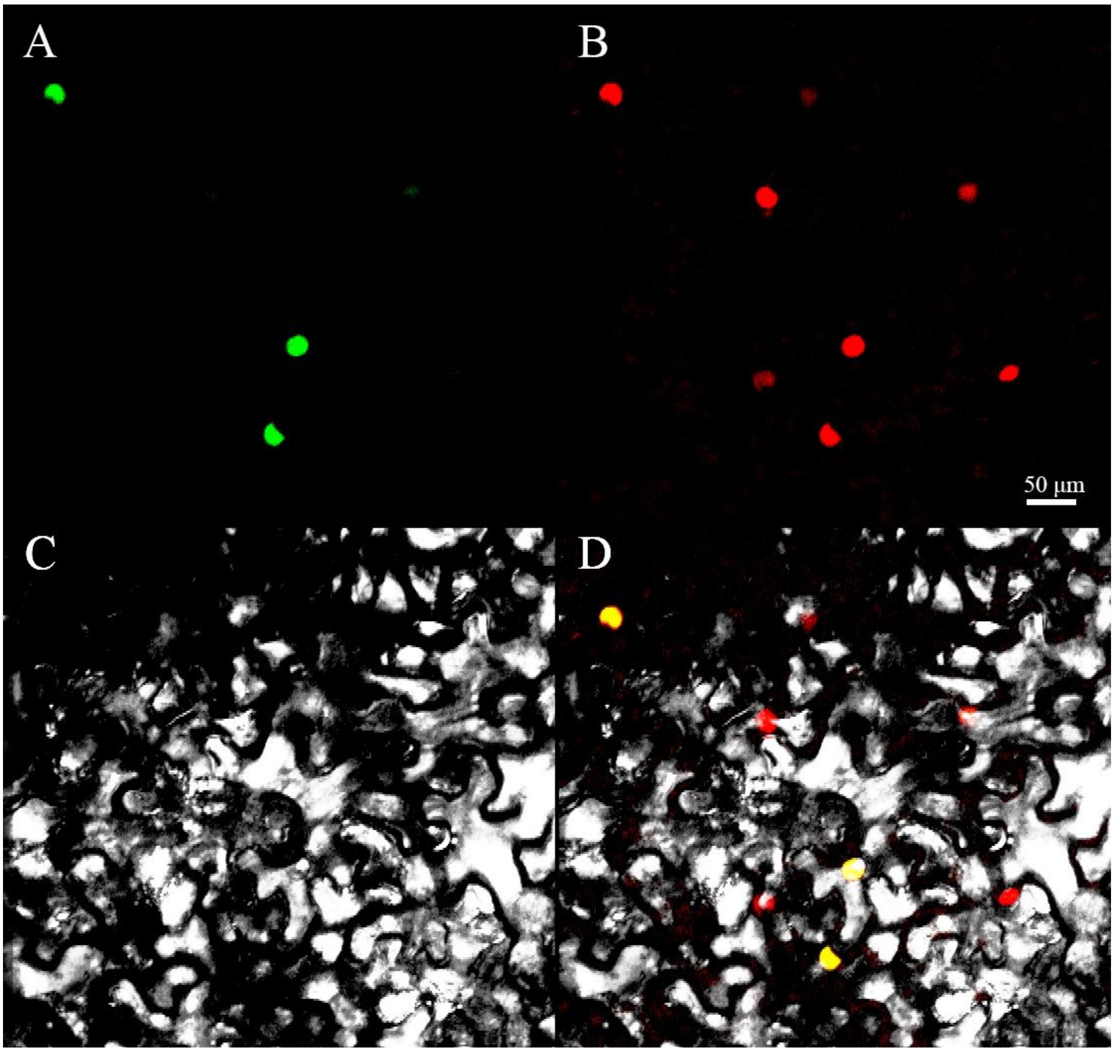

Fig. S6 Colocalization of MsNAC022-GFP fusion protein and the MsDREB6.2-RFP fusion protein in *N. benthamiana* leaf epidermal cells.

A. Confocal imaging of MsNAC022-GFP fusion protein in dark field. **B.** Confocal imaging of MsDREB6.2-RFP fusion protein in dark field; MsDREB6.2, a nuclear localization transcription factor[37]. **C.** Confocal imaging of *N. benthamiana* leaf epidermal cells in bright field; **D.** The mergence of A-C. Scale bar, 50 µm.

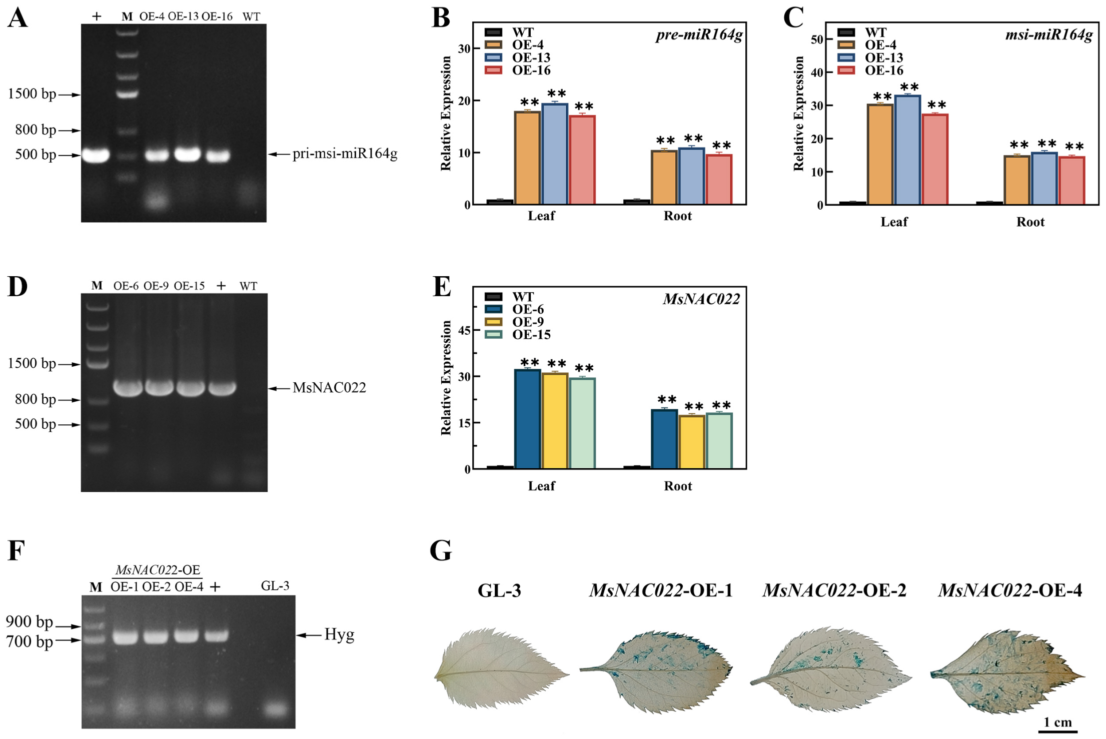

Fig. S7 Identification of *msi-miR164g*-OE and *MsNAC022*-OE transgenic plants.

A. Identification of *msi-miR164g*-OE transgenic Arabidopsis T_3 lines in DNA level. **B-C.** Transcriptional levels of the precursor of *msi-miR164g* (*pre-miR164g*) and *msi-miR164g MsNAC022* in transgenic Arabidopsis plants overexpressing *msi-miR164g*. Data are shown as means ± SD from three biological replicates. Asterisks indicate significant differences between the transgenic lines and WT (**$p < 0.01$, based on Duncan's multiple range test). **D.** Identification of *MsNAC022-OE* transgenic Arabidopsis T_3 lines in DNA level. **E.** Transcriptional levels of *MsNAC022* in transgenic Arabidopsis plants overexpressing *MsNAC022*. Data are shown as means ± SD from three biological replicates. Asterisks indicate significant differences between the transgenic lines and WT (**$p < 0.01$, based on Duncan's multiple range test). **F.** Identification of *MsNAC022*-OE transgenic 'GL-3' apple lines in DNA level; + : positive plasmid control; M: Marker III. **G.** Identification of *MsNAC022*-OE transgenic 'GL-3' apple lines by GUS staining. Scale bar, 1 cm.

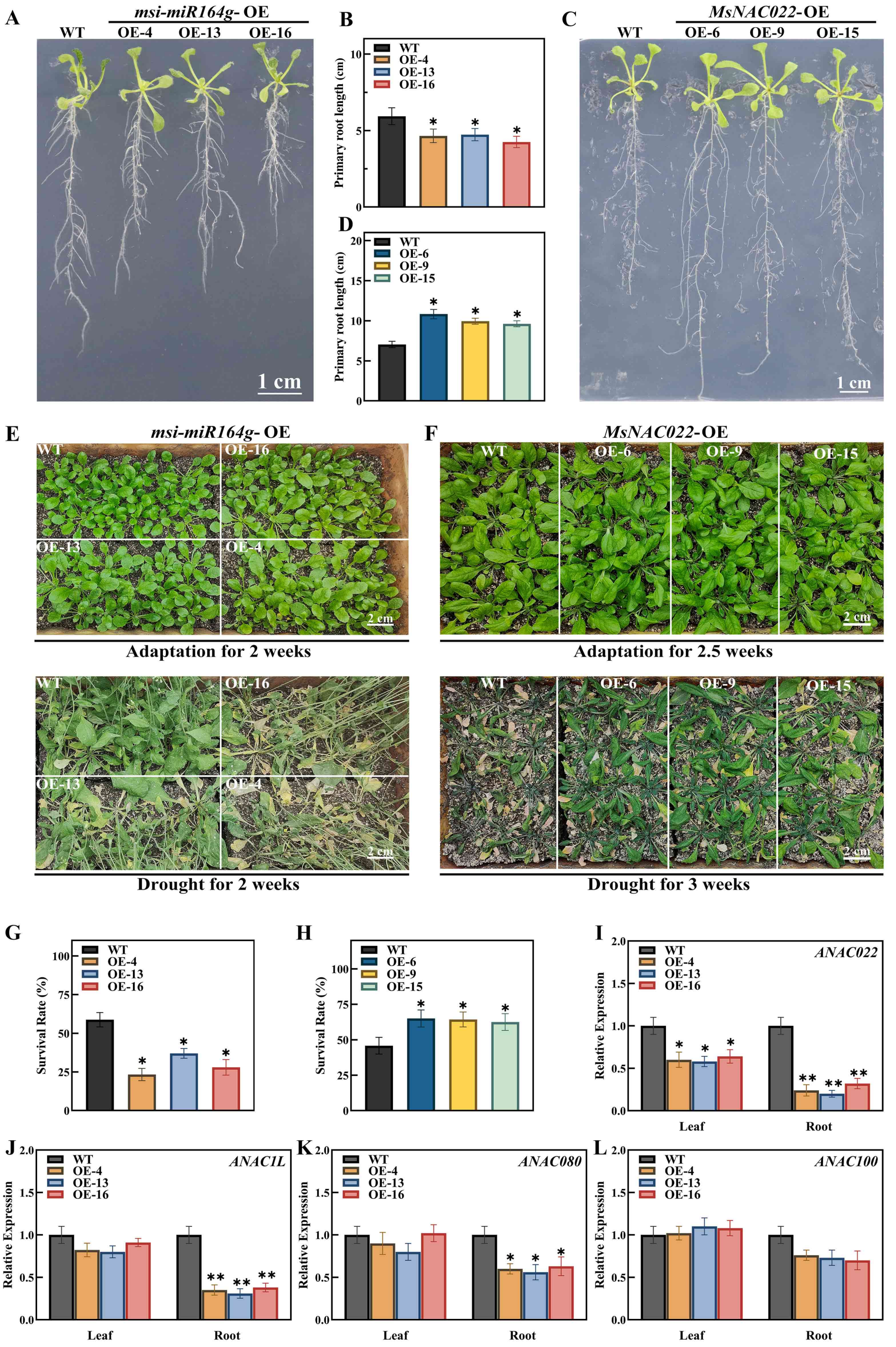

Fig. S8 The function of *msi*-miR164g-*MsNAC022* module in Arabidopsis.

A-D. Phenotypic characterization of primary root length in three independent Arabidopsis T_3 lines overexpressing *msi-miR164g* (OE-4, OE-13, OE-16) (A, B), *MsNAC022* (OE-6, OE-9, OE-15) (C, D) and the wild type (WT), respectively. **E-H.** Overexpression of *msi-miR164g* or *MsNAC022* in Arabidopsis alters plant resistance to drought stress. **I-L.** Transcript levels of the predicted target genes in transgenic Arabidopsis plants overexpressing *msi-miR164g*. Data are shown as means ± SD from three biological replicates. Asterisks indicate significant differences between the transgenic lines and WT (*$p < 0.05$, **$p < 0.01$, based on Duncan's multiple range test).

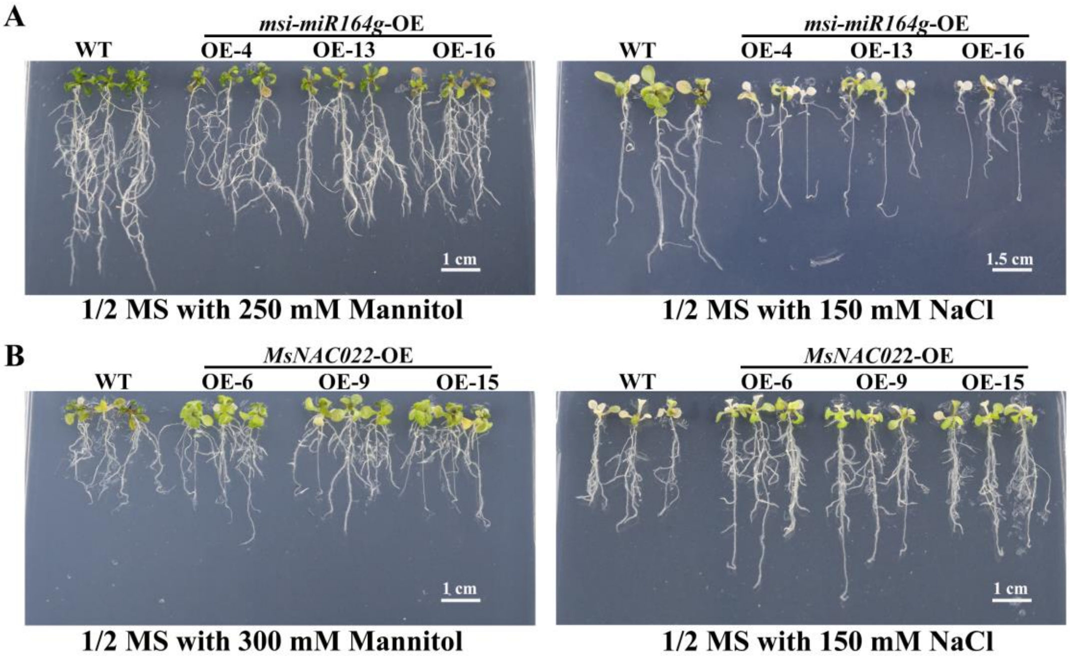

Fig. S9 Overexpression of *msi-miR164g* or *MsNAC022* in Arabidopsis alters plant resistance to osmotic and salt stress.

A. *msi-miR164g*-OE; **B.** *MsNAC022*-OE. Scale bar, 1 or 1.5 cm.

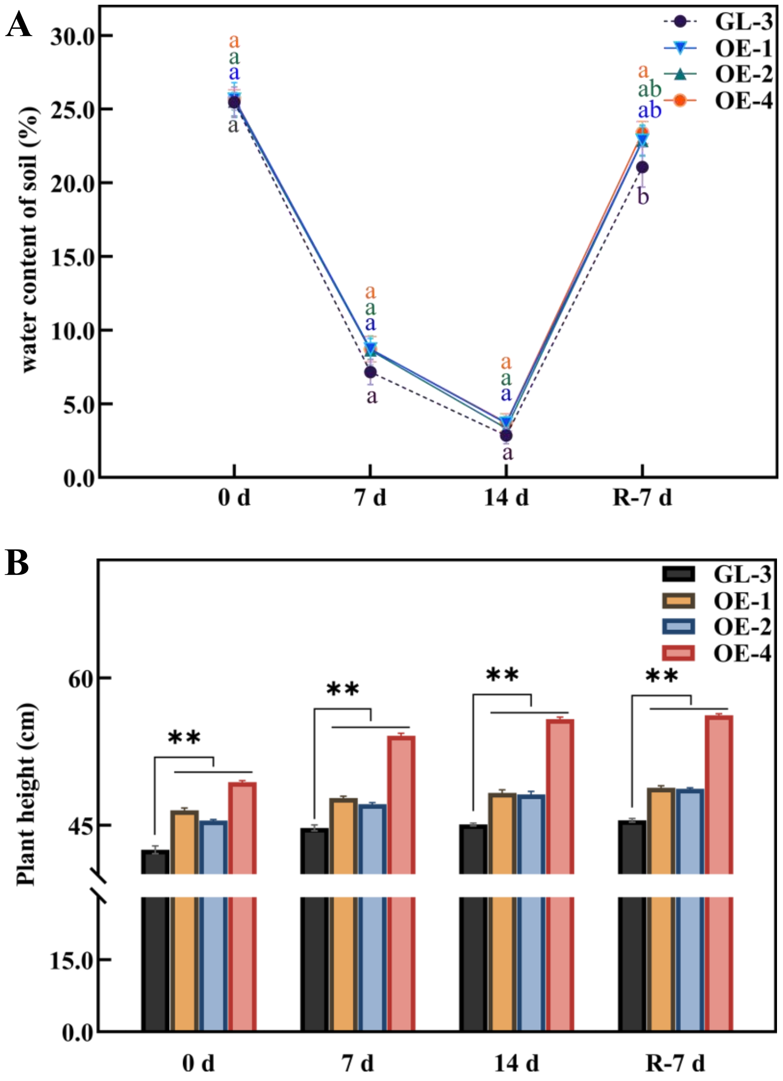

Fig. S10 Changes of soil moisture and the height of soil-grown from nontransgenic 'GL-3' and *MsNAC022*-OE apple plants after14 d drought stress and 7 d rewatering.

A. Soil moisture of different positions in the pot under drought stress. Data are means ± SD from three biological replicates. R-7d: rewatering for 7d. Different letters indicate significant differences between the transgenic lines and 'GL-3' plants ($p < 0.05$, based on Duncan's multiple range test). **B.** Plant height of the 5-month-old nontransgenic and transgenic apple plants after14 d drought stress and 7 d rewatering. Data are shown as means ± SD from three biological replicates. Asterisks indicate significant differences between the transgenic lines and 'GL-3' plants (*$p < 0.05$, **$p < 0.01$, based on Duncan's multiple range test).

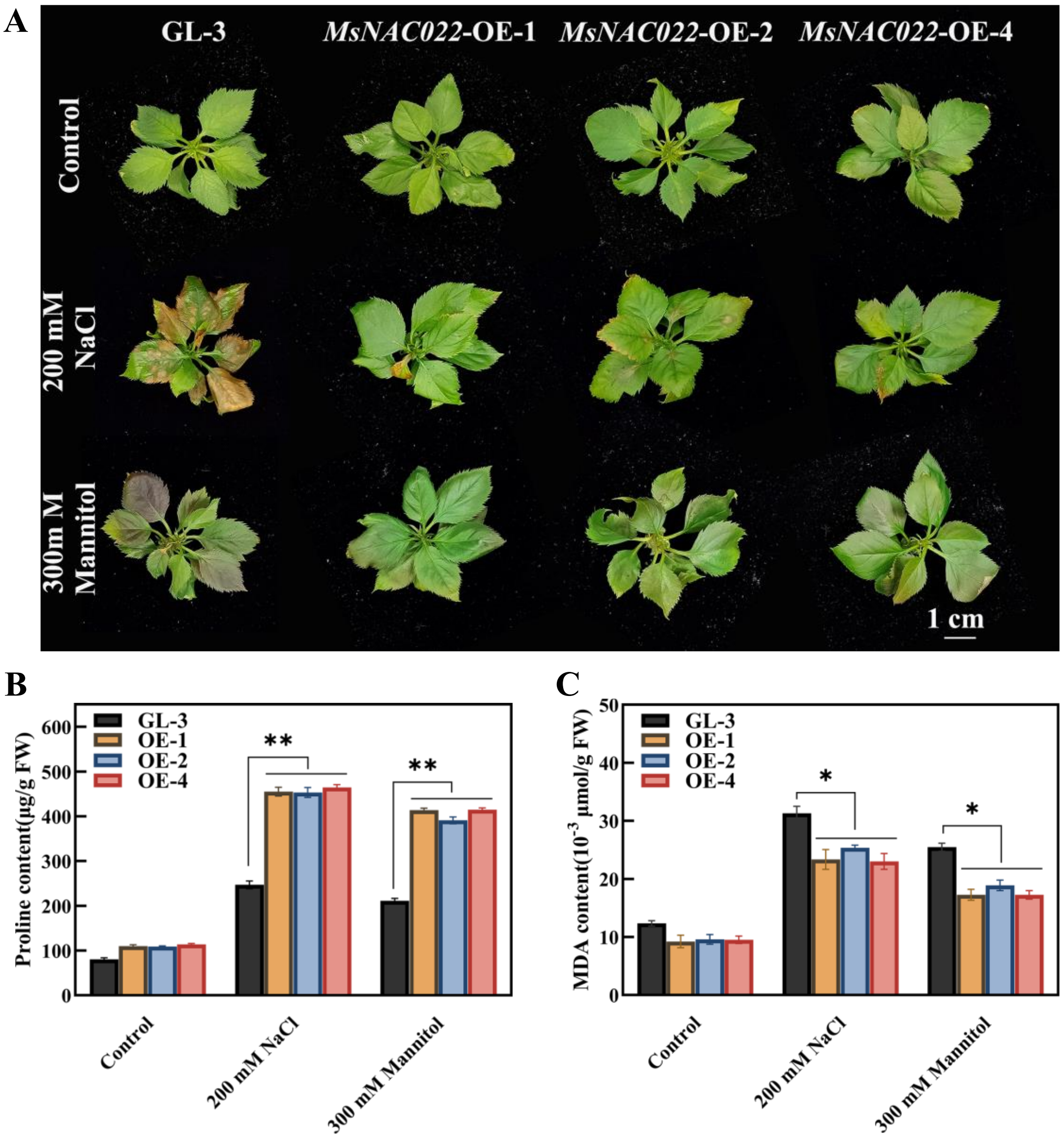

Fig. S11 Enhanced tolerance in transgenic apple plants to salinity and osmotic treatment. **A.** Phenotypic comparison of *MsNAC022*-OE apple plants to salt and osmotic stress in vitro. 30-d-old nontransgenic and transgenic apple plants were grown for 10 days under 200 mM NaCl or 300 mM mannitol treatment. Scale bar, 1 cm. **B-C.** Proline content (B) and MDA content (C) in nontransgenic and *MsNAC022* transgenic leaves subjected to salt and osmotic stress. Data are shown as means ± SD from three biological replicates. Asterisks indicate significant differences between the transgenic lines and 'GL-3' plants (*$p < 0.05$, **$p < 0.01$, based on Duncan's multiple range test).

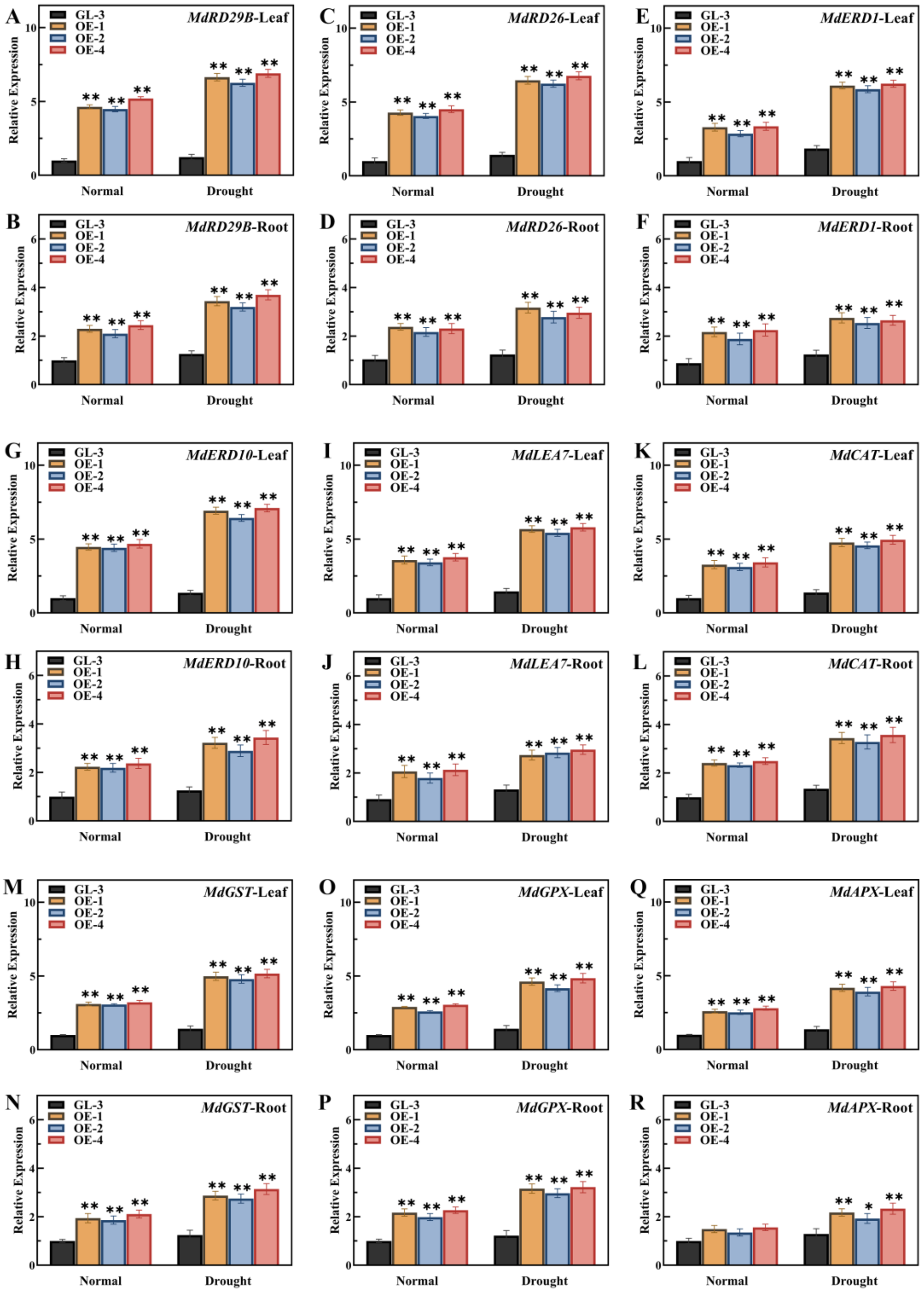

Fig. S12 Other drought stress-related genes and antioxidant enzyme related genes induced in *MsNAC022*-OE apple plants.

A-R. Expression levels of drought stress-related and ROS scavenging systems related genes in nontransgenic and transgenic apple plants under normal conditions and drought stress. A-B, *MdRD29B*; C-D, *MdRD26*; E-F, *MdERD1*; G-H, *MdERD10*; I-J, *MdLEA7*; K-L, *MdCAT*; M-N, *MdGST*; O-P, *MdGPX*; Q-R, *MdAPX*. Data are shown as means ± SD from three biological replicates. Asterisks indicate significant differences between the transgenic lines and 'GL-3' plants (*$p < 0.05$, **$p < 0.01$, based on Duncan's multiple range test).

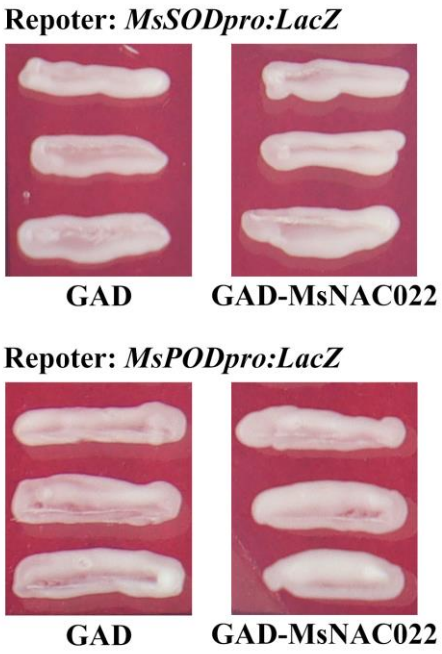

Fig. S13 Failure to detect direct binding between MsNAC022 and the *MsPOD* or *MsSOD* promoters in a yeast one-hybrid assay.

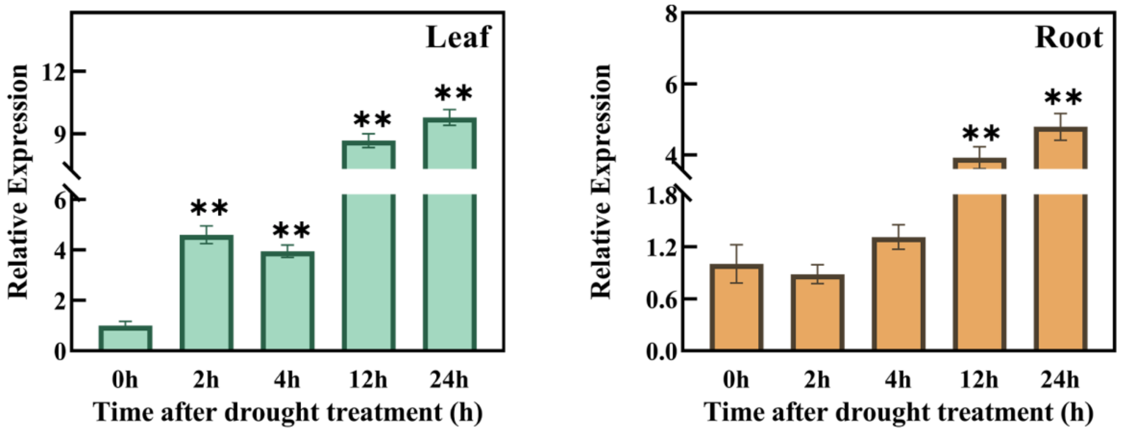

Fig. S14 Expression patterns of *MsPOD* in *M. sieversii* treated with 20% PEG 6000. Expression patterns of *MsPOD* in *M. sieversii* treated with 20% PEG 6000. Data are shown as means ± SD from three biological replicates. Asterisks indicate significant differences (*p < 0.05, **p < 0.01, based on Duncan's multiple range test).
